# Chronic activation of human cardiac fibroblasts in vitro attenuates the reversibility of the myofibroblast phenotype

**DOI:** 10.1038/s41598-023-39369-y

**Published:** 2023-07-26

**Authors:** Caitlin Hall, Jonathan P. Law, Jasmeet S. Reyat, Max J. Cumberland, Shaun Hang, Nguyen T. N. Vo, Kavita Raniga, Chris J. Weston, Christopher O’Shea, Jonathan N. Townend, Katja Gehmlich, Charles J. Ferro, Chris Denning, Davor Pavlovic

**Affiliations:** 1 Institute of Cardiovascular Sciences, University of Birmingham, Edgbaston, Birmingham, B15 2TT UK; 2 Department of Stem Cell Biology, Biodiscovery Institute, University of Nottingham, University Park, Nottingham, NG7 2RD UK; 3 Institute of Immunology and Immunotherapy, University of Birmingham, Edgbaston, Birmingham, B15 2TT UK; 4 Department of Cardiology, Queen Elizabeth Hospital, University Hospitals Birmingham NHS Foundation Trust, Edgbaston, Birmingham, B15 2GW UK; 5 Department of Renal Medicine, Queen Elizabeth Hospital, University Hospitals Birmingham NHS Foundation Trust, Edgbaston, Birmingham, B15 2GW UK

**Keywords:** Cardiovascular biology, Cardiovascular diseases, Heart stem cells, Mechanisms of disease, Cell biology

## Abstract

Activation of cardiac fibroblasts and differentiation to myofibroblasts underlies development of pathological cardiac fibrosis, leading to arrhythmias and heart failure. Myofibroblasts are characterised by increased α-smooth muscle actin (α-SMA) fibre expression, secretion of collagens and changes in proliferation. Transforming growth factor-beta (TGF-β) and increased mechanical stress can initiate myofibroblast activation. Reversibility of the myofibroblast phenotype has been observed in murine cells but has not been explored in human cardiac fibroblasts. In this study, chronically activated adult primary human ventricular cardiac fibroblasts and human induced pluripotent stem cell derived cFbs (hiPSC-cFbs) were used to investigate the potential for reversal of the myofibroblast phenotype using either subculture on soft substrates or TGF-β receptor inhibition. Culture on softer plates (25 or 2 kPa Young’s modulus) did not alter proliferation or reduce expression of α-SMA and collagen 1. Similarly, culture of myofibroblasts in the presence of TGF-β inhibitor did not reverse myofibroblasts back to a quiescent phenotype. Chronically activated hiPSC-cFbs also showed attenuated response to TGF-β receptor inhibition and inability to reverse to quiescent fibroblast phenotype. Our data demonstrate substantial loss of TGF-β signalling plasticity as well as a loss of feedback from the surrounding mechanical environment in chronically activated human myofibroblasts.

## Introduction

Cardiac fibrosis, both diffuse and focal, underlies multiple forms of cardiovascular disease (CVD) and is strongly associated with an increased risk of death^1–3^. A study by Gulati et al. found that patients with midwall fibrosis, linked to dilated cardiomyopathy, had increased risk of cardiovascular death and transplantation, sudden cardiac death and heart failure death^2^. Diabetes has also been linked to increased cardiac fibrosis and has been found to have increased incidence of hospitalisation for heart failure in these patients^4^. Furthermore, fibrosis has been linked to an increased susceptibility to arrhythmia and ventricular tachycardia^5^. There is, understandably, great interest in the development of effective therapeutic strategies which target reversal of cardiac fibrosis and have the potential to reduce morbidity and mortality^6–8^.

Cardiac fibroblasts comprise roughly 30% of the total myocardial cell population providing both an essential structural support and a communication network coupled by gap junctions^9,10^. Fibroblasts support normal cardiac structure and electromechanical function through homeostasis of the extracellular matrix (ECM) and cross-talk with other cardiac cell types^11^. Mechanical stress, inflammation, hormonal stimulus or ischaemia lead to activation of cardiac fibroblasts and excessive deposition of ECM, with an increase in collagen cross linking. This cardiac fibrosis leads to remodelling of the myocardium adversely affecting function, initially in diastole, and conduction^12,13^.

Pathological cardiac remodelling is associated with activation and differentiation of fibroblasts into myofibroblasts^14^. Myofibroblasts are characterised as having a spindle shape, expression of contractile protein α-smooth muscle actin (α-SMA), secretion of ECM components such as collagens and a change in proliferation rates^15,16^. Transition of fibroblasts into myofibroblasts occurs following immediate or sustained injury, which is mediated through several processes, including increased mechanical stress and secretion of cytokines, such as transforming growth factor-beta (TGF-β). Several studies have demonstrated that TGF-β activates cardiac fibroblasts^17–19^, and contributes to in vitro and in vivo murine cardiac fibrosis and hypertrophy^20,21^. Further studies utilising models of TGF-β inhibition or knockdown have shown an attenuated fibrotic response. However, perhaps unsurprisingly, due to the complexity and widespread involvement of the TGF-β signalling pathway increased mortality in murine models has also been observed suggesting this might not be a successful target for therapeutic use^22,23^. In order to identify more practicable targets, there is a need for additional models, in particular, those utilising human cells.

Healthy human myocardium is described as having a Young’s modulus of ~ 10–30 kPa. Diseased or damaged myocardium is much stiffer, at more than 100 kPa, often due to the presence of fibrosis^24–27^. Recent studies have shown that use of stiff substrates (> 50 kPa), which mimic the elastic modulus of a fibrotic myocardium, leads to activation of cardiac fibroblasts and transition into myofibroblasts^28,29^. Studies utilising murine cardiac fibroblasts demonstrate that this transition begins within days of isolation and culture, even in the absence of TGF-β^30,31^. It has therefore been suggested that the use of softer substrates, which more closely resemble the elastic modulus of healthy myocardium may prevent and possibly reverse activation of cardiac fibroblasts when maintained in 2D culture^25,30^. Whether once activated, human myofibroblasts can be transformed back to the quiescent cardiac fibroblast phenotype is not known.

Given the central role of the myofibroblast in fibrosis, this study investigates the potential for reversal of activated human cardiac myofibroblasts back to quiescent fibroblasts, as a means to reduce the fibrotic burden of the heart. Our data demonstrate that chronically activated human myofibroblasts cannot be reversed back to their quiescent, non-activated, phenotype, both in adult primary human cardiac fibroblasts and human induced pluripotent stem cell derived cardiac fibroblasts.

## Materials and methods

### Human cardiac fibroblast culture

Human cardiac fibroblasts (HCF) obtained from the ventricles of explanted hearts taken at the time of cardiac transplantation were purchased from PromoCell GmbH (C-12375) and received cryopreserved at passage 2. The donors were aged 60 and 63. These cells were used as a model of chronically-activated myofibroblasts as found in chronic heart failure. These HCFs were thawed and cultured using Fibroblast Growth Medium 3 (PromoCell®, C-23025) for up to 10 passages (split ratio 1:10) in T75 tissue culture flasks and kept in a humidified incubator at 37 °C and 5% CO_2_. These cells were used for experimentation between P5 and P10 and kept in culture for roughly 4 weeks. HCFs were cultured on stiff polystyrene culture plates (Young’s modulus [*E*] = 3 GPa) from Corning before subculture on soft (*E* = 2 kPa and 25 kPa) culture plates (Matrigen Softwell, SW6-EC-2/SW6-EC-25). Matrigen Softwell culture plates achieve lower Young’s modulus than standard polystyrene or glass cultureware through a transparent, two-dimensional layer of bisacrylamide crosslinked polyacrylamide hydrogel along the entire cell culture surface. The product is supplied sterile and tissue-culture treated. Standard cell culture procedures such as cell culture media, dissociation with TryPLE Express (Thermo Fisher Scientific, 12,604,013) and cell culture dish area (9.6 cm^2^ per well) were kept constant across all tissue cultureware during experiments. In order to promote efficient adhesion of HCFs, all cultureware were coated with 10 μg/mL fibronectin (Sigma-Aldrich, F1141). Fibronectin is produced by cardiac fibroblasts and is a principal component of the cardiac ECM^32,33^.

**Figure 1:**
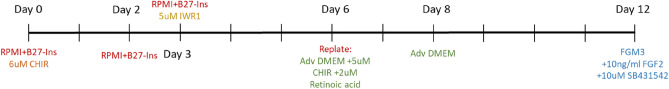
Protocol for derivation of hiPSC-cFbs using CHIR99021 (GSK-3 inhibitor), IWR-1-endo (Wnt/beta-catenin inhibitor), Retinoic acid and Fibroblast Growth Factor 2^34^. SB431542 is a TGF-β receptor type 1 inhibitor.

The human induced pluripotent stem cell (hiPSC) line Kolf2-C1, derived from the skin of a healthy, male British Caucasian individual aged 55–59, was used to generate hiPSC-derived cardiac fibroblasts (hiPSC-cFbs) using an established protocol (Fig. 1)^34^. Day 0 cells were exposed to RPMI + B27 –Insulin supplemented with 6 µM CHIR99021 (Tocris Bioscience, 4424) for 48 h. Media was changed to fresh RPMI + B27 –Insulin at day 2. Day-2 hiPSCs were then seeded at 30,000 cells/cm^2^ in Essential 8 medium (Lifetech, A1517001) on Matrigel® (Corning®, 356,231). At day 3, cells were supplemented with 5 µM IWR1 (Sigma, I0161) for 72 h. On day 6, cells were replated at a density of 20,000 cells/cm^2^ in Advanced DMEM supplemented with 5 µM CHIR99021 and 2 µM Retinoic Acid. Cells were media changed with Advanced DMEM 48 h later and left for 4 days. On day 12, cells were replated using Fibroblast Growth Medium 3 (FGM3) in the presence of 10 µM SB431542 (Selleck Chemicals, S1067) and 10 ng/ml Fibroblast Growth Factor 2 (FGF2) (Peprotech, 100-18B). hiPSC-cFbs were maintained in FGM3 supplemented with SB431542 and FGF2 and cultured on Matrigel® for up to 6 passages (split ratio 1:6).

### In vitro treatments

The following drugs were added to the respective culture media as summarised in Table 1: 10 ng/mL recombinant human TGF-β1 protein expressed from a CHO cell line (R&D Systems, 240-B)^35^, 30 nM TGF-β receptor 1 inhibitor SD208 (Tocris, 3269)^36^, 10 μM TGF-β receptor inhibitor SB431542^34^.

**Table 1:** Experimental combinations and outcomes for HCFs and iPSC-cFbs.

Cell type	Substrates (*E*)	Drug treatments	Treatment duration	Outcome
HCF	3 GPa, 25 kPa, 2 kPa	10 ng/ml TGF-β, 30 nM SD208, 10 μM SB431542	2–4 days	Proliferation: CyQUANT; RT-PCR: α-SMA, collagen 1; IF: α-SMA; western blot: α-SMA, collagen 1
hiPSC-cFb	3 GPa	10 ng/ml TGF-β, 30 nM SD208, 10 µM SB431542	2–4 days	Proliferation: CyQUANT; RT-PCR: α-SMA, collagen 1, collagen 3; IF: α-SMA

*α-SMA* alpha-smooth muscle actin, *E* elastic modulus, *HCF* human cardiac fibroblast, *IF* immunofluorescence, *hiPSC*-*cFb* human induced pluripotent stem cell-derived cardiac fibroblast, *TGF*-*β* transforming growth factor-beta, *RT*-*PCR* real-time reverse transcriptase polymerase chain reaction.

### CyQUANT® NF proliferation assay

Cells were seeded in 96 well plates at 7500 cells/cm^2^ (HCF) or 15,000 cells/cm^2^ (hiPSC-cFbs) in the presence or absence of 10 ng/ml TGF-β (ED_50_ 0.04–0.2 ng/ml)^37^, 30 nM SD208 or 10 µM SB431542. Cells were then left for 3–4 h to adhere (Day 0) at 37 °C and 5% CO_2_ or were left for 48 h (Day 2). The CyQUANT® NF proliferation assay (Invitrogen™, C35006) was used according to the manufacturer’s protocol and fluorescence measured using the FlexStation® 3 microplate reader (Molecular Devices). A percentage change was calculated by comparing Day 2 data with Day 0 data.

### Immunofluorescence microscopy

Cells were fixed with 4% paraformaldehyde at room temperature (RT) for 10 min. Cells were then permeabilised with 0.1% Triton™ X-100 for 5 min at RT and then blocked with 5% bovine serum albumin (BSA) for 1 h at RT. Cells were then incubated with primary antibodies overnight at 4 °C. Antibody raised against vimentin (abcam®, ab45939) was used at a concentration of 0.25 µg/ml and the antibody directed towards α-SMA (Sigma-Aldrich, A2547) at a dilution of 1:750 (HCFs) or 1:500 (hiPSC-cFbs). The cells were then blocked for a second time with 5% BSA for 30 min at RT before being incubated with secondary antibodies (abcam®, Alexa Fluor® 488, ab150077 or Thermofisher Scientific, Alexa Fluor® 647, A21235) at 1:1000 dilution for 90 min at RT. Cells were then incubated with 1:1000 DAPI for 30 min before mounting with Hydromount™. Cells were visualised using the Zeiss LSM780 confocal laser scanning microscope equipped with the objective C-Apochromat 40x/1.20 W. Images were acquired using the Zen Black software (version 3.0). For the purpose of data acquisition and analysis, all acquisition settings were kept the same. Relative fluorescence intensity was calculated for each cell using ImageJ by dividing raw integrated density by cell area.

### mRNA expression by RT-PCR

Total RNA extraction from cells was performed using the RNeasy Mini Kit (Qiagen, 74106). The quantity and purity of RNA eluted was determined with a NanoDrop™ Spectrophotometer (Thermo Scientific, ND-2000). Single-stranded cDNA was synthesised from RNA using the High-Capacity cDNA Reverse Transcription Kit (Applied Biosystems™, 4368814) according to manufacturer protocols.

Real-time reverse transcriptase polymerase chain reaction (RT-PCR) was performed using TaqMan™ gene expression chemistry (Table 2). Samples were run in triplicate. GAPDH was used as an endogenous housekeeping gene and inter-plate calibrator. Samples with GAPDH C_T_ values greater than two standard deviations from the mean C_T_ of the dataset were excluded from analysis. Relative gene expression values were determined by 2^–ΔΔCT^ method using vehicle-treated cells for comparison.

**Table 2:** TaqMan gene expression assays.

Gene	Protein	Assay ID
*ACTA2*	α-Smooth muscle actin	Hs00426835_g1
*COL1A1*	Collagen type 1	Hs00164004_m1
*COL3A1*	Collagen type 3	Hs00943809_m1
*GAPDH*	Glyceraldehyde 3-phosphate dehydrogenase	Hs02786624_g1

### Western blotting

Cell pellets were re-suspended in 50 µL RIPA buffer (Sigma-Aldrich, R0278) with 1% Halt™ Protease and Phosphatase Inhibitor Cocktail 100X (Thermo Scientific, 78441) and incubated on ice for 30 min. Protein concentration was determined by DC™ protein assay (Bio-Rad, 5000116) prior to dilution in 2X sample loading buffer and incubation at 95 °C for 5 min.

Samples (up to 20 ug per lane) were subjected to SDS-PAGE on 4–15% precast gels (Bio-Rad, 4561086). Proteins were transferred onto PVDF membranes using Trans-Blot Turbo™ Mini Transfer Packs and Transfer System (Bio-Rad, 1704156/1704150). Membranes were blocked in 5% milk containing tris-buffered saline with 0.1% Tween 20 (Bio-Rad, 1610734/1710781) before probing with primary and secondary antibodies as listed in Table 3.

**Table 3:** Western blotting antibodies.

Target	Company, catalog#	Host	Concentration
Glyceraldehyde 3-phosphate dehydrogenase	CST, D16H11	Rabbit	1:10,000
α-Smooth muscle actin	CST, D4K9N	Rabbit	1:1000
Collagen 1	Abcam, ab34710*	Rabbit	1:1000
Rabbit IgG	Li-Cor 800CW 926–32211	Goat	1:10,000

*ab34710 anti-collagen 1 antibody has affinity to both α1 and 2 subunits. Two bands are seen at ~ 130 and 170 kDa. Both subunits were quantified and expressed as a total expression relative to GAPDH loading control.

Visualisation and quantification of protein band density was performed using the Odyssey Fc imaging System and Image Studio Lite v5.2 (Li-Cor) without any post-capture image manipulation. GAPDH was used as loading control; the band intensity of the protein of interest (POI) was normalised to the GAPDH loading control and presented as a ratio – POI/GAPDH – to account for protein loading inconsistencies.

### Statistical methods

Data are presented as mean ± standard error of the mean. Differences between group means were examined using nested one-way analysis of variance (ANOVA) or Student’s *t* test and were considered statistically significant when P < 0.05. Subgroups for the nested analysis were replicates done on different days with different passage numbers. Statistical analysis was conducted using GraphPad Prism 9.

## Results

### Long-term culture of human cardiac fibroblasts leads to myofibroblast transition

Myocardium stiffness has been associated with activation of cardiac fibroblasts to myofibroblasts^24^. Culture of primary healthy murine cardiac fibroblasts on stiff plastic plates is shown to induce myofibroblast activation^30^. HCFs were obtained from the explanted hearts of patients undergoing cardiac transplantation and cultured in a 2D monolayer on “stiff” polystyrene plates (E = 3 GPa) for up to 10 passages in order to chronically activate HCFs. These cells were then exposed to 10 ng/mL TGF-β, a well-established inducer of the myofibroblast phenotype, for 2–4 days to assess their ability to further activate—2 days for immunofluorescence and proliferation experiments (to avoid high confluency) and 4 days for qPCR and western blotting. α-SMA and collagen 1 were utilised as markers of fibroblast activation and transition to myofibroblasts. Vimentin was used as a marker for cardiac fibroblasts.

**Figure 2:**
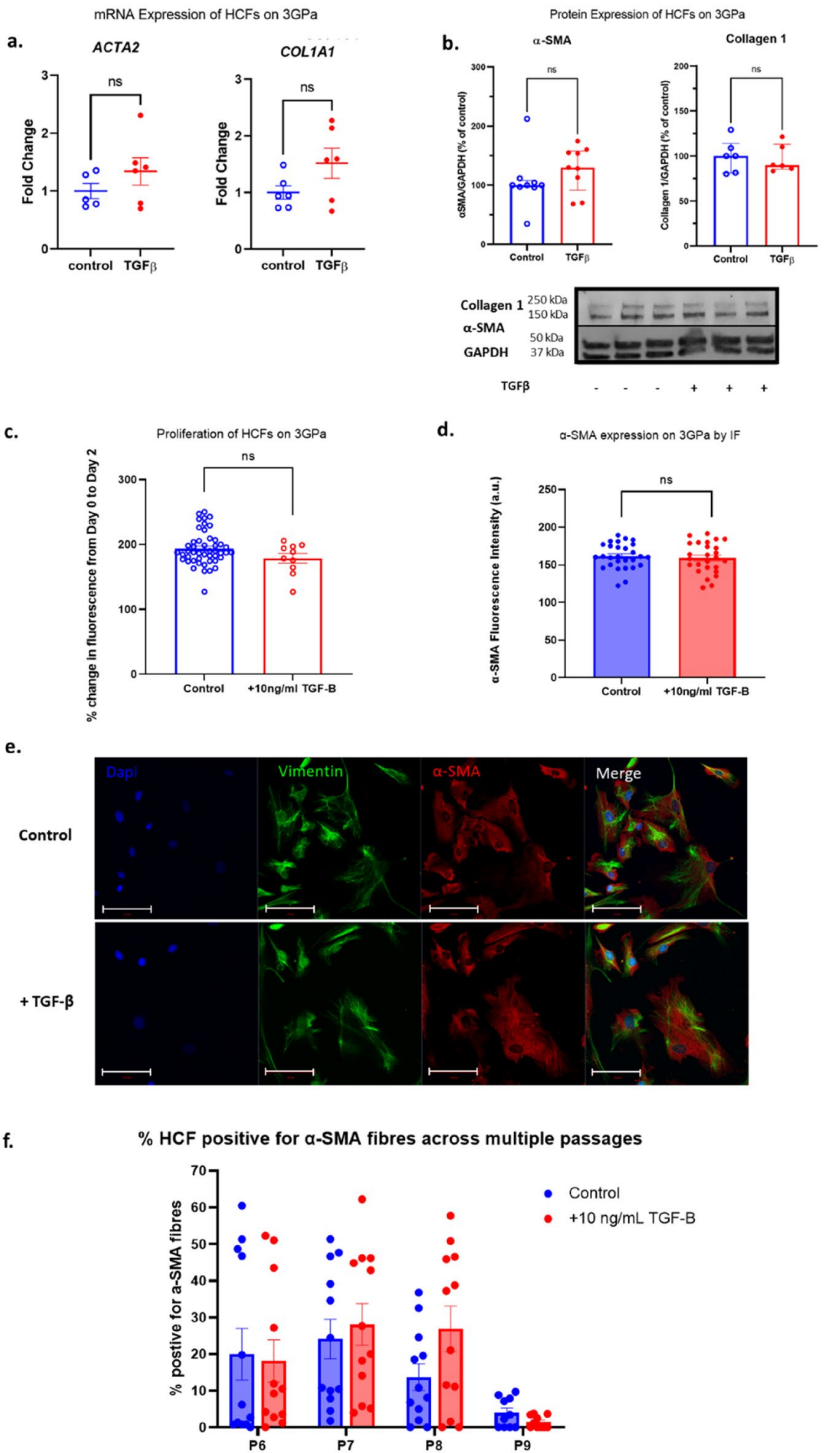
Activation status and proliferation of human cardiac fibroblasts on 3 GPa culture plates. Cells were grown in the presence and absence of 10 ng/ml TGF-β. (**a**) Activation of HCF as measured by RT-PCR for ACTA2 and COL1A1. (**b**) Activation status of HCF measured by western blotting for α-SMA and collagen 1 protein. (**c**) Proliferation over 48 h was evaluated as a percentage change from baseline (day 0) in fluorescence by CyQUANT assay. (**d**) Quantification of α-SMA protein expression by immunofluorescence. (**e**) Representative immunofluorescence images for figure (**d**). Scale bar = 100 µm. (**f**) percentage of HCF positive for α-SMA fibrous staining at P6-9 followed by exposure to 10 ng/mL TGF-β for 2 days. Data presented as mean ± S.E.M. N = 5 (**a**), 6–9 (**b**); 4 plates, 6 + wells per plate (**c**), 3 experiments, 3 images per condition (**d**), 6 wells, 2 images per well, percentage cells per image (**f**). *t* test (**a**,**b**) and nested one-way ANOVA (**c**,**d**) compared with undrugged control. Significance was defined by p < 0.05.

Following long term culture on stiff substrates, α-SMA was detected by western blot and immunofluorescence staining in HCF cultured on 3 GPa polystyrene plates indicating baseline activation (Fig. 2, panels b, d and e). Treatment with TGF-β for 4 days did not lead to significant increase in α-SMA and Collagen 1 expression at the mRNA level (Fig. 2a) or protein level (Fig. 2b) when compared to vehicle control. TGF-β also had no detectable effect on proliferation (Fig. 2c) or cell size (Fig. S1). ~ 70% of HCFs displayed positive staining for α-SMA when assessed by immunofluorescence (Fig. S2).

TGF-β treatment did not significantly affect α-SMA expression, as assessed by immunofluorescence (Figs. 2d and f, Fig. S2). Secondary antibody only controls show that this was not due to non-specific binding or background fluorescence staining (Fig. S3). This lack of TGF-β-induced response was maintained at each passage tested post thaw (p6-p9), when assessed by immunofluorescence (Fig. 2f and Fig. S4a). Total α-SMA expression as measured by relative integrated density did not significantly change from passage 6 to 9 (Fig. S4a). Similarly, percentage of cells positive for α-SMA fibres did not significantly change during long term culture from p6-p9 (Fig. 2f). Interestingly, long term culture indicated a non-significant decrease % of α-SMA positive cells (p =  > 0.05) with long term culture indicating possible de-differentiation of HCF.

These results clearly demonstrate that standard 2D culture on stiff plastic of human cardiac fibroblasts, isolated from diseased hearts, leads to persistent myofibroblast transition and renders them unresponsive to TGF-β stimulation.

### Chronically activated human fibroblasts cannot transition back to quiescent phenotype

We then investigated whether the established myofibroblast phenotype can be reversed. Two approaches for myofibroblast de-activation were employed, via reduction in mechanical stress (using sub-culture on softer substrates, 25 and 2 kPa) or via TGF-β receptor inhibition (using small molecule inhibitor SD208).

**Figure 3:**
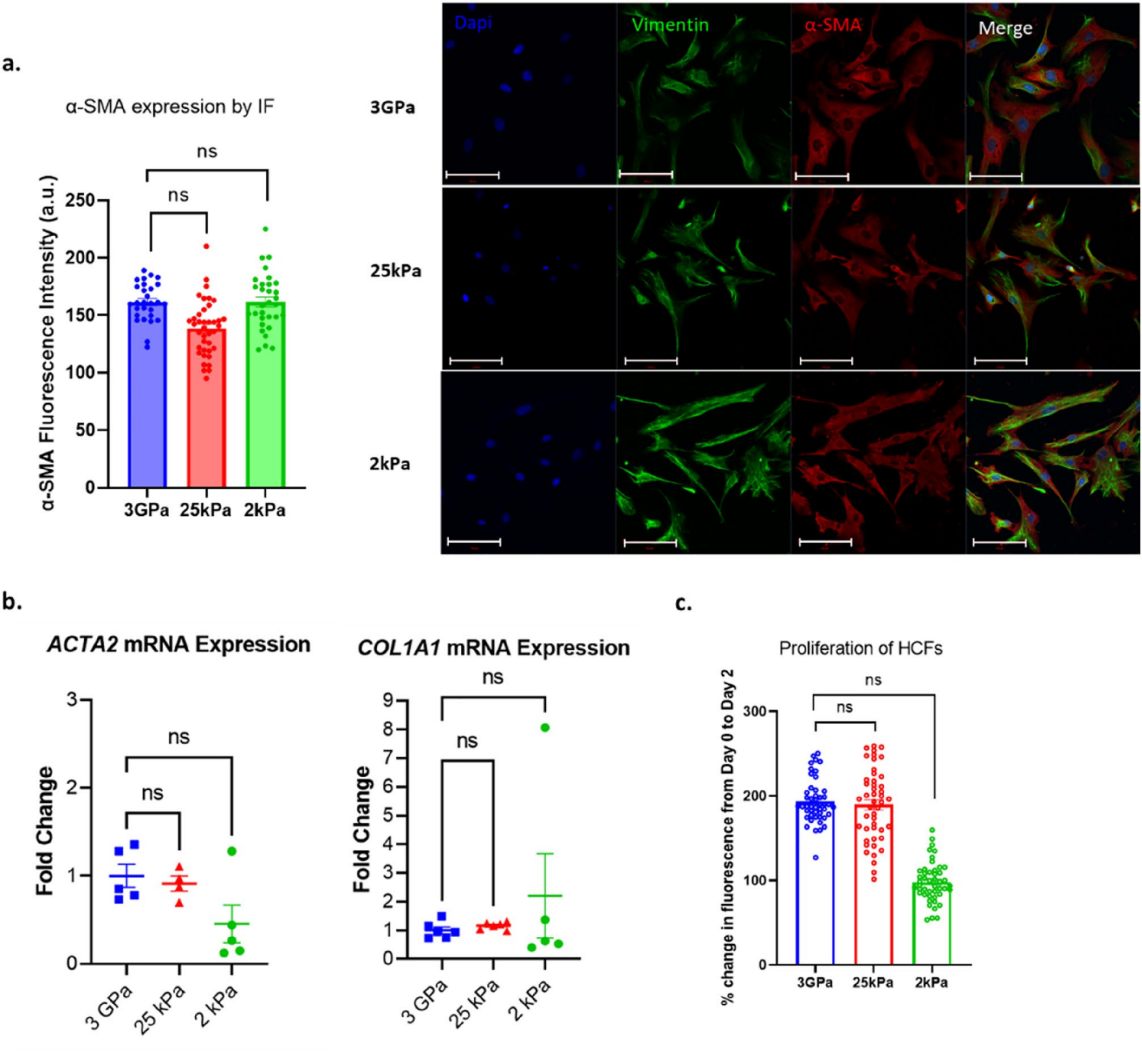
Activation status and proliferation of human cardiac fibroblasts on 3 GPa, 25 and 2 kPa culture plates. (**a**) Vimentin and α-SMA expression by immunofluorescence after 2 days in culture. Nuclei (DAPI in blue), vimentin (green), α-SMA (red) Scale bar = 100 µm. (**b**) α-SMA (ACTA2) and Collagen 1 (COL1A1) expression by RT-PCR following 4 days in culture. (**c**) Proliferation was assessed over 48 h and evaluated as a percentage change in fluorescence from baseline (day 0). Data presented as mean ± S.E.M. N = 3 experiments, 3 images per condition (**a**), 4–6 (**b**); 4 plates, 47 wells per plate (**c**). Nested one-way ANOVA compared with control (3GPa cells). Statistical significance was defined as p < 0.05.

Culture on 25 kPa or 2 kPa soft substrates did not result in significant reduction in α-SMA expression in myofibroblasts, as measured by immunofluorescence (Fig. 3a). These findings were supported by RT-PCR analysis of *ACTA*2 and *COL1A1* mRNA transcripts, which showed comparable levels of mRNA expression between myofibroblasts cultured on the stiff and soft substrates (Fig. 3b). When proliferation was assessed over a 48-h period, no significant difference was seen between myofibroblasts grown on 3 GPa and 25 kPa (Fig. 3c). A trend towards non-significant reduction in proliferation of myofibroblasts grown on 2 kPa when compared with 3 GPa was observed (Fig. 3c). Cell size was unchanged on 25 kPa (Fig. S1) but significantly decreased for myofibroblasts grown on 2 kPa substrates (Fig. S1) compared with 3 GPa.

TGF-β receptor inhibitor SD208 (30 nM) had no discernible effect on proliferation of myofibroblasts grown on 3 GPa (Fig. S5). There was also no significant effect on α-SMA expression, as measured by immunofluorescence, following treatment with SD208 (Fig. S5b). Similarly, percentage of cells positive for α-SMA was also unchanged in the presence of SD208 (Fig. S2).

We next assessed whether a dual approach of myofibroblast deactivation, via both subculture on soft substrates and in the presence of TGF-β inhibitors, can reverse myofibroblasts to their quiescent phenotype. Culture of myofibroblasts on 25 kPa substrate, in the presence of SD208, demonstrated no significant effect on proliferation (Fig. S6c), nor change in expression of α-SMA, as assessed by immunofluorescence (Fig. S6d and e). As demonstrated in Fig. S6, mRNA and protein expression of α-SMA and Collagen 1 were also unaffected by addition of TGF-β, when cultured on 25 kPa (Fig. S6a and b, summarised in Table 4).

**Table 4:** Myofibroblast markers on soft culture plates by RT-PCR and western blot.

RT-PCR	Gene of interest	TGF-β mean fold change versus control	P	
25 kPa	*ACTA2*	1.2 ± 0.6	0.9	
	*COL1A1*	1.0 ± 0.1	0.9	
2 kPa	*ACTA2*	0.4 ± 0.2	0.6	
	*COL1A1*	0.5 ± 0.1	0.8	

Western blot	Protein of interest	Control (POI/GAPDH)	TGF-B (POI/GAPDH)	P
25 kPa	α-smooth muscle actin	107.2 ± 6.8	168.7 ± 26.5	0.2
	Collagen 1	101.3 5.5	97.8 7.1	0.7
2 kPa	α-smooth muscle actin	96.6 ± 3.5	130.2 ± 29.4	0.3
	Collagen 1	142.2 ± 52.8	135.9 ± 53.8	0.9

Data presented as mean ± S.E.M. *t* test of TGF-β treated cells versus vehicle control. Western blot data presented as a ratio of protein of interest (POI) and GAPDH as a loading control.

Culture on 2 kPa in the presence of SD208 similarly yielded no significant differences in proliferation (Fig. S7c), nor expression of α-SMA (Fig. S7d and e). Expression of α-SMA and Collagen 1 were also unaffected at the mRNA transcript or protein level (Fig. S7a and b, summarised in Table 4).

To examine whether longer-term TGF-β receptor inhibition is required to reverse chronically activated HCF, myofibroblasts were cultured from p5 to p9 in the presence or absence of TGF-β receptor inhibitor SB431542 (SB). At each passage, α-SMA expression was assessed by immunofluorescence. Total α-SMA expression as measured by relative integrated density did not significantly change from passage 6 to 9 (Fig. S4a). Similarly, % cells positive for α-SMA fibres did not significantly change compared to myofibroblasts cultured in the presence of SB (Fig. S4b). Our data also demonstrate that HCF are unresponsive to TGF-β at each passage tested (p6-p9) regardless of whether they are cultured in the presence or absence of SB (Fig. S4c).

These data show that myofibroblasts cannot be de-activated when subsequently cultured on softer substrates (25 kPa or 2 kPa) that more closely reflect that of the human myocardium. These data further demonstrate that human myofibroblasts do not respond to TGF-β treatment and cannot transition back to quiescent state when cultured on soft substrates, in the presence or absence of a TGF-β inhibitor.

### Chronically activated hiPSC derived cardiac fibroblasts cannot be reversed to a quiescent phenotype

**Figure 4:**
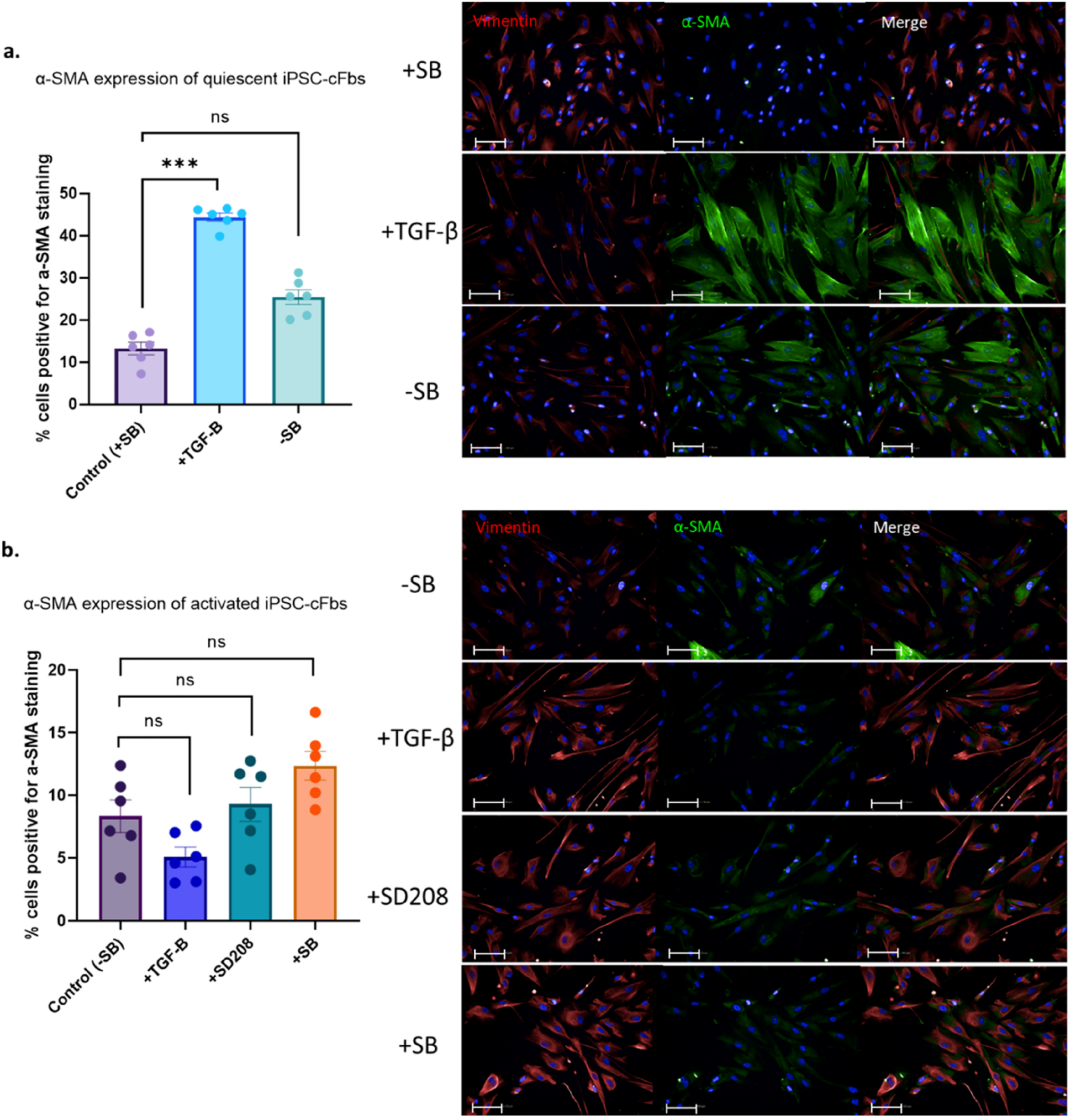
Immunofluorescence staining of α-SMA in quiescent and activated human induced pluripotent stem cell derived cardiac fibroblasts (hiPSC-cFbs). (**a**) Quiescent hiPSC-cFbs generated through culture for 2 weeks in the presence of SB435142 were treated with TGF-β. The effect of withdrawal of SB435142 (SB) was assessed. (**b**) Activated hiPSC-cFbs generated through culture for 2 weeks in the absence of SB435142 were also treated with TGF-β. In addition, the effect of TGF-β receptor inhibition with SD208, and SB435142 (SB) re-introduction was assessed. Data presented as percentage of cells displaying α-SMA expression by immunofluorescence. Scale bar 100 µm. Data presented as mean ± S.E.M. *N* = 3 plates, 18 wells, nested one-way ANOVA compared with control.

To further investigate whether chronic activation of human cardiac fibroblasts is reversible, separate experiments using human iPSC-cFbs were performed (see Fig. S8 for protocol schematic). hiPSC-cFbs were cultured on 3 GPa substrates in the presence of TGF-β receptor inhibitor SB431542 to generate a quiescent cardiac fibroblast or in the absence of SB431542 to generate an activated fibroblast (myofibroblast) phenotype. Quiescent cells showed a significant increase in α-SMA expression when exposed to TGF-β (Fig. 4a). Of note is that after 2 days of culture on stiff surfaces in the absence of TGF-β a two-fold increase in the number of quiescent cells positively expressing α-SMA was observed (Fig. 4a), indicating that culture on stiff substrates alone induces α-SMA expression.

As expected, exposure to TGF-β, or subsequently to inhibitors SD208 and SB431542, did not significantly modify α-SMA expression in activated hiPSC-cFbs, when compared to the control (Fig. 4b). This mirrors our observation with the activated HCFs; culture on stiff substrates (3 GPa) activates cardiac fibroblasts and makes them unresponsive to TGF-β stimulation and inhibition. In line with our previous observations (see Sect. “Long-term culture of human cardiac fibroblasts leads to myofibroblast transition”), culture of hiPSC-cFbs over 2 weeks in the absence of SB431542 reduced the number of α-SMA positive cells that display fibre-like staining pattern (see Fig. 4b).

An additional qualitative assessment of α-SMA expression was conducted over the period of 1 week using immunofluorescence. hiPSC-cFbs were cultured in the absence of a TGF-β receptor inhibitor. This data confirms that iPSC-cFbs are activated by culture on 3 GPa substrates alone, independent of TGF-β, albeit to a lesser extent (Figs. S9 and S10). This data also shows that activation by hard substrates begins within 24 h, with a peak at around 96 h of culture (Figs. S9 and S10).

**Figure 5:**
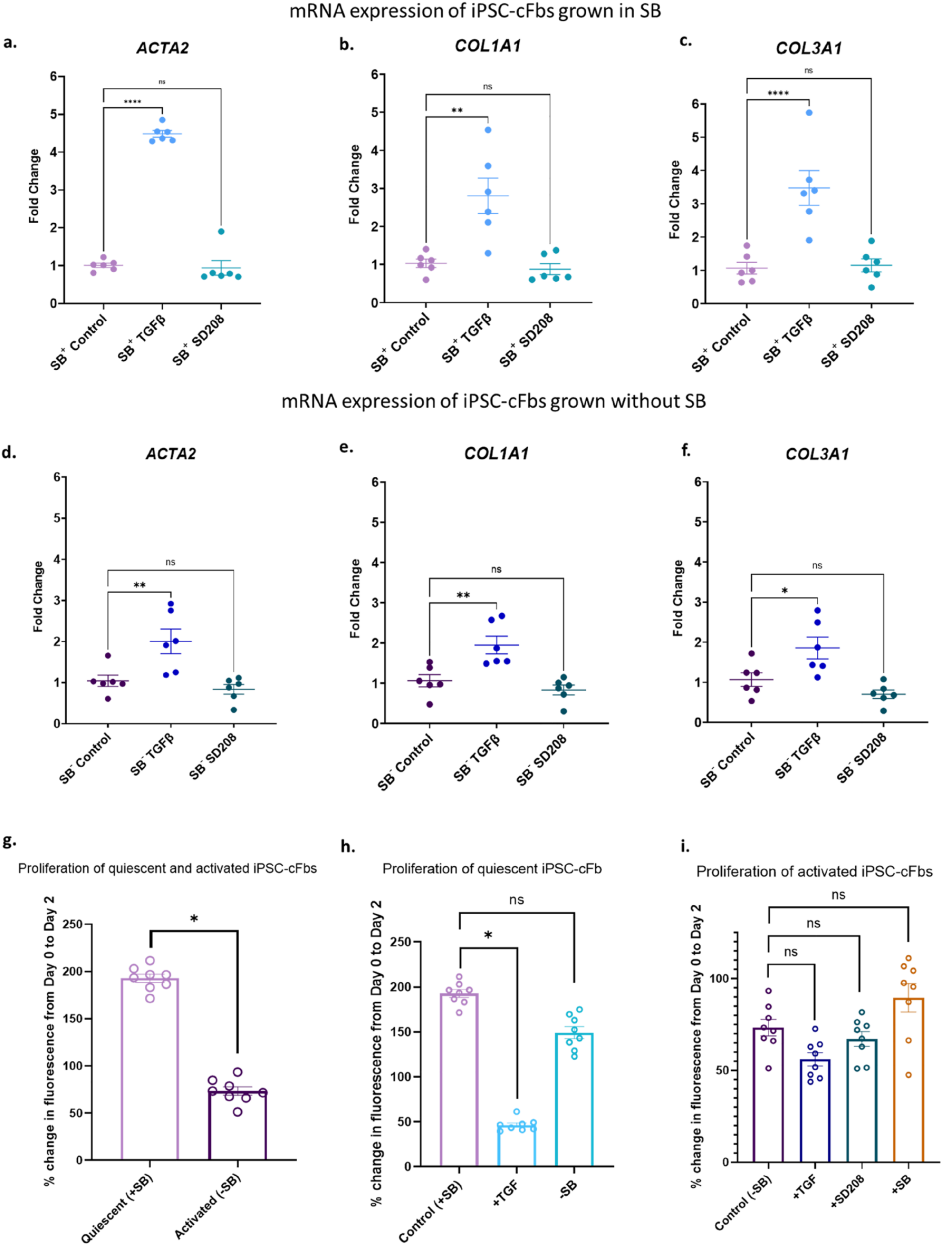
Activation status of quiescent and activated human induced pluripotent stem cell derived cardiac fibroblasts (hiPSC-cFbs) in response to TGF-β and SD208. (**a**-**f**) Activation of quiescent and activated hiPSC-cFbs as determined by RT-PCR for *ACTA2, COL1A1 and COL3A1* following 4 days in culture. Data presented as mean ± S.E.M. *N* = 6. *p < 0.05, **p < 0.01, ****p < 0.0001, one-way ANOVA compared with control. (**g**–**i**) Proliferation was assessed as a percentage change in fluorescence over 48 h compared to baseline. (**g**) Proliferation of untreated quiescent and activated hiPSC-cFBs. (**h**) Proliferation of quiescent hiPSC-cFBs in the presence of TGF-β (+ TGF) and following withdrawal of SB431542 (-SB). (**i**) Proliferation of activated hiPSC-cFBs in response to TGF-β (+ TGF) and TGF-β receptor inhibitors SD208 (+ SD208) and SB431542 (+ SB). Data presented as mean ± S.E.M. N = 3 plates, 24 wells. *p < 0.05, nested one-way ANOVA compared with control.

Characterisation of mRNA expression changes of hiPSC-cFbs in response to TGF-β by RT-PCR demonstrated that quiescent hiPSC-cFbs displayed a robust increase in α-SMA, collagen 1 and collagen 3 versus vehicle treated hiPSC-cFbs (Fig. 5a–c). Activated hiPSC-cFbs also had an observable but attenuated response to TGF-β, leading to a comparatively smaller increase in α-SMA, collagen 1 and collagen 3 (Fig. 5d–f). SD208 treatment did not alter the expression of α-SMA, Collagen 1 or Collagen 3 in either the activated or quiescent hiPSC-cFbs (Fig. 5).

Proliferation of hiPSC-cFbs was also assessed. Activated hiPSC-cFbs show a significantly reduced proliferation rate when compared to their quiescent counterparts (Fig. 5g). These data suggest that in hiPSC-cFbs, activation leads to a reduction in proliferation, rather than the expected increase in proliferation.

Quiescent hiPSC-cFbs demonstrated higher proliferation rates compared to the activated hiPSC-cFbs (Fig. 5g). A decrease in proliferative capacity was observed in response to TGF-β in quiescent hiPSC-cFbs (Fig. 5h). The proliferative capacity of quiescent hiPSC-cFbs showed a small but non-significant decrease when SB431542 was removed from the culture media, (Fig. 5h). Activated hiPSC-cFbs did not respond to either recombinant TGF-β or inhibition of the TGF- β signalling pathway by SB431542 or SD208 (Fig. 5i).

These results mirror that seen in the primary HCFs, demonstrating that chronically activated myofibroblasts cannot be reversed to a quiescent phenotype using TGF-β receptor inhibition.

## Discussion

There have been significant efforts to identify effective therapeutic approaches against long-established cardiac fibrosis targets. However, clinical trials of drugs which target the mineralocorticoid receptor, renin-angiotensin system, natriuretic peptide degradation, vascular tone, and others have yielded mixed results in reducing fibrosis-related outcomes in patients with heart failure with preserved and reduced ejection fraction and ischaemic heart disease despite encouraging preclinical data (Reviewed in^38^). Given the central role of the myofibroblast in cardiac fibrosis, our study intended to investigate the potential for the reversal of activated human cardiac myofibroblasts to a more quiescent phenotype.

The principal findings of this study (Table 5) are that chronically activated primary human cardiac ventricular fibroblasts show a diminished response to activation by TGF-β and that neither culture on softer substrates, nor TGF-β receptor inhibition are able to reverse the observed myofibroblast phenotype. Our findings are confirmed in hiPSC-cFbs, indicating that chronic activation of human cardiac fibroblasts leads to a terminal differentiation into myofibroblasts. The lack of response to TGF-β was not associated with a change in expression of the TGF-β receptor itself (Fig. S11). We further demonstrate that culture of hiPSC-cFbs on stiff surfaces results in activation within 24 h, and intriguingly longer-term culture (> 96 h) results in the loss of fibrous α-SMA expression.

**Table 5:** Summary table of principal findings.

Cell type	Pre-treatment conditions	Treatment conditions	Outcomes	
			Proliferation	Myofibroblast markers
HCFs from patients with diabetic cardiomyopathy	Long-term culture on stiff substrates (3 GPa, ≤ 10 passages)	Stiff substrate (3 GPa) ± TGF-β ± TGF-βRi	No change	No change
		Soft substrate (25 kPa, 2 kPa) ± TGF-β ± TGF-βRi	No change	No change
hiPSC-cFbs	Quiescent hiPSC-cFbs (2 week culture *with* TGF-βRi)	TGF-β	Reduced	Increased
		TGF-βRi	No change	No change
	Activated hiPSC-cFbs (2 week culture *without* TGF-βRi)	TGF-β	No change	No change
		TGF-βRi	No change	No change

*hiPSC*-*cFbs* human induced pluripotent stem cell derived cardiac fibroblasts, *TGF*-*β* transforming growth factor-beta, *TGF*-*βRi* transforming growth factor-beta receptor inhibitor.

### Proliferative capacity of human cardiac fibroblasts

Conflicting data exists concerning the correlation between cardiac fibroblast activation and proliferation. Intriguingly, our data highlights significantly higher proliferation rates in quiescent hiPSC-cFbs compared to the activated hiPSC-cFbs. Accordingly, TGF-β treatment reduced the proliferative capacity in quiescent hiPSC-cFbs but did not alter proliferation of activated hiPSC-cFbs or fibroblasts isolated from a patient. Similarly, Hecker et al. also showed that human lung myofibroblasts, activated using TGF-β, exhibited less proliferation than quiescent fibroblasts as well as an attenuated proliferative response to serum^19^. When using freshly isolated mouse cardiac fibroblasts, Herum et al. observed no changes in proliferation rates when cultured on multiple different substrates (3, 8, 10, and 50 kPa) as well as no changes when substrates were stiffened from 3 or 8 kPa to 30 kPa after 5 days of culture^29^. A further study using human cardiac fibroblasts found that although there was a higher density of vimentin positive cells in failing hearts, there was a significant decrease in the proliferative marker Ki-67 in the failing hearts when compared to the non-failing hearts. Myofibroblasts (α-SMA positive cells) from both groups showed similarly low levels of Ki-67 staining^22^. Other studies have implicated the renin–angiotensin–aldosterone system in the proliferation of cardiac fibroblasts^39,40^. Using freshly isolated rat cardiac fibroblasts, Olson et al. were able to attenuate myofibroblast differentiation and cardiac fibroblast proliferation by inhibiting mitogen-activated protein kinase mediated signalling with polyphenolresveratrol^41^. Whether the same affects would be observed in vivo remains to be studied.

Cardiac fibroblasts derive from mesenchymal cells with a variety of origins. These include, but are not limited to, the pro-epicardium, epicardium and cardiac endothelium through epithelial- or endothelial- to mesenchymal transformation (EMT and EndMT, respectively). Although cardiac fibroblasts show a decreased proliferative capacity once having undergone myofibroblast differentiation, cellular cues may instigate enhanced EMT/EndMT to maintain or increase cardiac fibroblast numbers within the heart^42,43^. The exact processes underpinning this require further investigation, where targeted attenuation of these mechanisms could present an alternative avenue to decreasing the fibrotic burden of the heart.

### Reversal of myofibroblast phenotype through TGF-β inhibition

Cardiac fibrosis, whilst generally thought of as a maladaptive and pathophysiological process, is essential to ensuring tissue integrity following acute injury. For example, fibrotic tissue replaces necrotic myocardium following myocardial infarction thus maintaining cardiac mechanical function and preventing myocardial rupture^17^. This replacement fibrosis leads to impaired systolic and diastolic function, and to arrhythmias, likely around its margins. In contrast, diffuse interstitial fibrosis appears to be a purely maladaptive response with adverse effects on both ventricular function and susceptibility to arrhythmia^44^. The key role of TGF-β in cardiac fibroblast activation and proliferation is well-studied. Clinical studies have consistently reported upregulated TGF-β expression in the myocardium of patients with heart failure^45–47^. The induction of fibrosis because of activation of myofibroblasts via small mothers against decapentaplegic (SMAD) and non-SMAD-mediated signalling pathways such as ERK, JNK and p38 has also been previously described^24,48,49^.

A recent study by Nagaraju et al. similarly investigated the potential for reversal of cardiac fibroblast activation using TGF-β receptor inhibition. Using freshly isolated human cardiac fibroblasts from explanted hearts of transplant recipients, the authors found that incubation with the TGF-β receptor inhibitor SD208 resulted in loss of α-SMA and reduced expression of profibrotic genes^22^. To achieve this, a high concentration of SD208 (3 µM) was utilised. In contrast, we found no effect on the activation status (proliferation rate and α-SMA expression) of isolated human ventricular cardiac fibroblasts when SD208 was used at a concentration of 30 nM (IC_50_ = 49 nM^50^), similar to that used in other cardiac fibroblast studies^36^.

Inhibition of TGF-β or its signalling components has been shown to be an effective anti-fibrotic treatment in preclinical studies. Transgenic rodent studies have demonstrated that disruption of fibroblast-specific TGF-β receptor 1 produced cardiac-protective effects against fibrosis and diastolic dysfunction^35^. Further in vivo studies utilising neutralising monoclonal antibodies and small molecular inhibitors directed against TGF-β and TGF-β receptor 1, respectively, reported significant reductions in cardiac fibrosis in murine tissue^23,51,52^. However, anti-TGF-β therapies are associated with significant dose-limiting drug toxicity given the ubiquitous expression of TGF-β and its receptors in healthy tissue throughout the human body, which restricts the effectiveness of such treatments. Mitra et al. reported widespread dose-dependent complications associated with pan-TGF-β inhibition in both mouse and monkey models. These included vascular and cardiac valvular inflammation, systemic bleeding, bone dysplasia and increased mortality^53^. Studies employing targeted inhibition via small molecular inhibitors against TGF-β receptor 1 also reported similar cardiac and haemorrhagic complications^54,55^. Due to these challenges, there has yet to be an anti-TGF-β therapy that has successfully found the balance between achieving an adequate anti-fibrotic response whilst avoiding serious cardiovascular side effects, despite candidate drugs such as galunisertib showing initial promise in cancer trials^56,57^.

### Terminally differentiated cardiac myofibroblasts cannot be de-activated

The discrepancy between our reported findings of irreversibly activated myofibroblasts and that of Nagaraju et al.^22^ may also be explained by differing cell culture approaches, specifically, the duration in culture and passage number prior to application of a de-activating agent or treatment. In their experiments, the authors utilised the isolated cardiac fibroblasts within 4 days of isolation without passaging, in contrast to our HCF and hiPSC-cFb cell lines, which were intentionally cultured long-term for up to 10 passages/2–4 weeks before usage to produce a chronically activated myofibroblast phenotype. Taken together, our results suggest the presence of a ‘threshold’ after which fibroblasts become unresponsive to a de-activating treatment, which warrants further discussion.

Studies have supported the concept that the plasticity of myofibroblast phenotype is only conserved and demonstrable when using freshly isolated cells^25^, or cells which have been cultured in ‘activating’ conditions for a short period of time^58^. Tomasek and Gabbiani^59^ challenged the idea of the fibroblast and myofibroblast being discrete, categorical phenotypes. Instead, they proposed the presence of an intermediate ‘protomyofibroblast’ stage, encompassing a continuum of cellular processes and fibroblast phenotypes, which bridge the differentiation of fibroblasts into myofibroblasts^60^. In parallel, the potential for myofibroblast de-differentiation would also follow a similar principle and depend on where they lie on the continuum of phenotypes. Indeed, data from single cell RNA sequencing already provides evidence that there are several distinct subpopulations of cardiac fibroblasts in healthy hearts^9,10^, and at different stages of ischaemic injury in murine studies^61,62^.

Our immunofluorescence data show that chronically activated HCFs and hiPSC-cFbs exhibited an unusual α-SMA staining pattern. Though these cells are unresponsive to TGF-β, and therefore assumed to be activated, they exhibit less of the “classic” actin fibres as found in our quiescent hiPSC-cFbs following TGF-β treatment. Instead, the activated cardiac fibroblasts displayed dispersed α-SMA staining within the cytoplasm (Fig. 2e, Fig. 3a and Fig. 4b). Use of secondary antibody-only controls shows that this effect is not due to non-specific staining (Fig. S3). The exact explanation for this is unclear, however we hypothesise that chronic culture in activating conditions may result in disassembly of actin fibres. This hypothesis may be supported by the decrease in α-SMA fibrous staining seen in Figs. S9 and S10 from 96 h to 1 week of activation by both hard substrates alone and in conjunction with TGF-β, as well as in Fig. S4 with a decrease from passage 6 to passage 9. The localisation of α-SMA within the cardiac fibroblast requires further investigation as a potential marker for late stage and/or irreversible activation status.

It is therefore conceivable that our cell culture approach, which bears resemblance to the persistent, longer duration of cardiac fibroblast activation at the latter stages of cardiac disease, resulted in irreversible terminal differentiation of myofibroblasts, a phenomenon that has been reported in other organ systems such as the lung^58,63^.

### The role of mechanical stiffness and myofibroblast activation

Our data show that human cardiac fibroblasts are activated by culture on stiff ~ 3 GPa substrates^58,63^. Chronically activated cardiac fibroblasts failed to transition back to a quiescent phenotype by subsequent culture on softer culture substrates (2 and 25 kPa), suggesting a loss of feedback from the surrounding mechanical environment. Healthy myocardium has a typical elastic modulus of between 10 and 30 kPa^25,64,65^, rising to over 100 kPa in diseased tissues^26,66^. Several other studies have indicated that prolonged culture on a hard plastic surface is sufficient to cause increased α-SMA expression in freshly isolated murine cardiac fibroblasts^40,65,67–69^. However, the cellular mechanisms that regulate the cardiac fibroblast phenotype and function in response to mechanical stimuli are complex. Studies have reported some success in de-activating human and porcine myofibroblasts through the use of soft hydrogels^25,70^ and three-dimensional microfabricated tissues associated with reduced tissue tension^71^. Still, the reversal tended to be incomplete, and experiments utilised freshly isolated cells cultured for shorter periods^40,69^, further supporting the concept of there being a range of protomyofibroblast states.

We also note that recommended culture of hiPSC-cFbs requires use of TGF-β receptor antagonist SB431542 at high concentrations (10 µM; IC_50_ 94 nM) to maintain quiescence^34,36^. It may be that use of soft substrates would allow the use of lower concentrations of TGF-β antagonism. In particular, the consequences of longer-term cardiac fibroblast culture in the presence of an inhibitor, and whether the effects of mechanical stress of the substrate can be fully suppressed over prolonged periods remain unclear.

## Conclusion and future direction

The lack of efficacious treatments for the reversal of cardiac fibrosis is a product of the complex cellular pathways, which are common to extracellular matrix generation and turnover in both health and disease. The cardiac fibroblast is the principal cell type in the fibrotic process and we show, using diseased patient-derived cardiac fibroblasts, that their activation into myofibroblasts under chronic disease conditions represents a challenging phenotype to reverse in vitro. Our experiments using hiPSC-cFbs largely recapitulated these findings. Whether cardiac fibroblasts isolated from healthy human myocardium would yield similar findings presents an intriguing and important avenue for future investigation.

## Supplementary Information

Supplementary Figures.

Supplementary Information.

## Data Availability

The datasets generated during and/or analysed during the current study are available from the corresponding author on reasonable request.
