# Supplementary material for: Chronic activation of human cardiac fibroblasts in vitro attenuates the reversibility of the myofibroblast phenotype
Source: Sci Rep. 2023 Jul 26;13:12137. doi: 10.1038/s41598-023-39369-y (PMC10372150; doi:10.1038/s41598-023-39369-y)
Supplement: Supplementary file 1 — Supplementary Figures. [file 41598_2023_39369_MOESM1_ESM.docx]

**
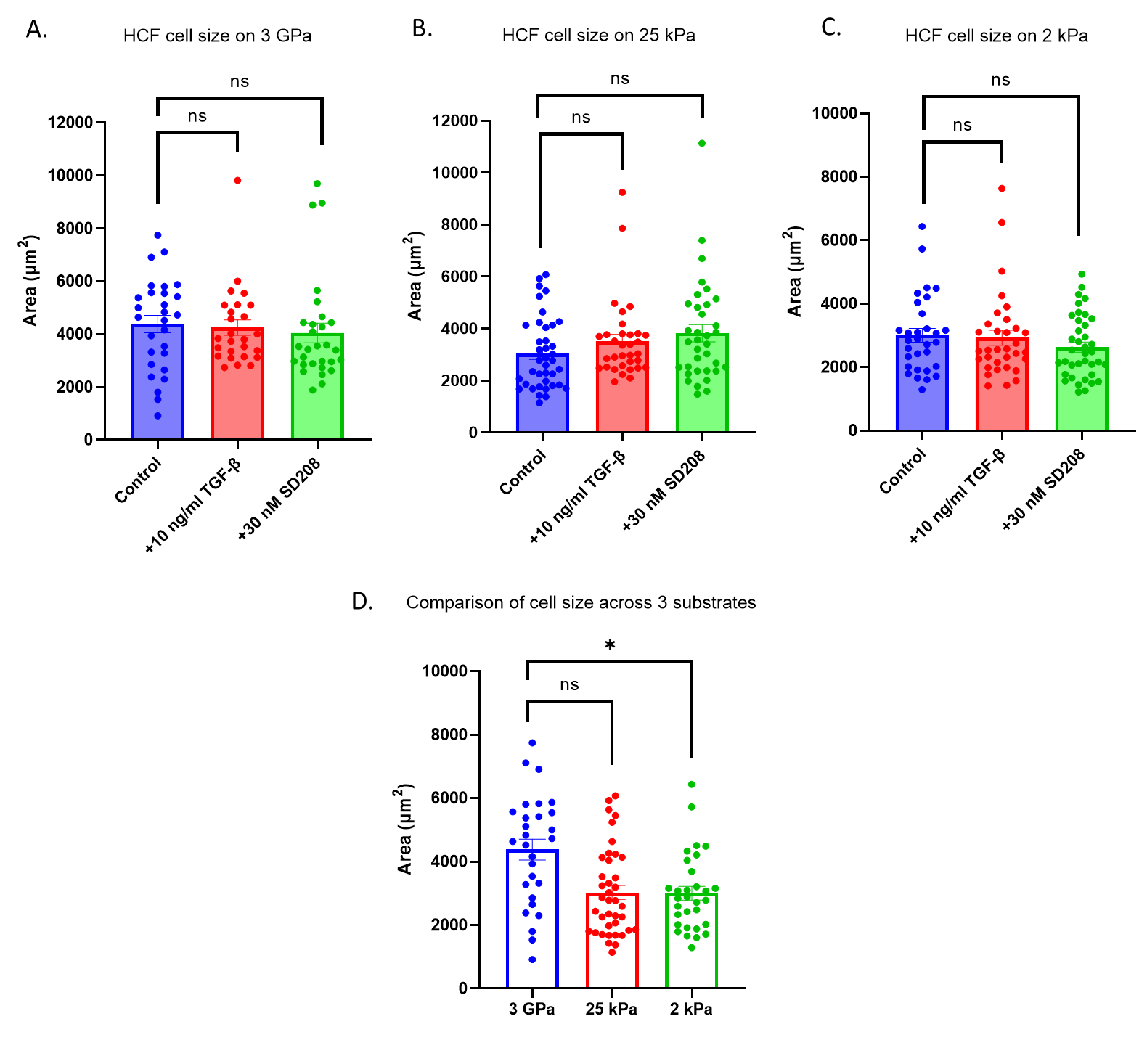
Supplementary figures**

Figure S1. Comparison of cell size for HCFs cultured on 3GPa, 25 kPa and 2 kPa assessed using cell area. A-C. Addition of TGF-β or SD208 had no significant effect on cell size on any of the three substrates. D. Culture on 2 kPa caused a significant decrease in cell size when compared to 3 GPa control (p = < 0.05).

Data presented as mean ± S.E.M. N = 3 wells, nested one-way ANOVA compared with control.

Figure S2. Immunofluorescence staining of α-SMA in HCFs. Cells were grown in the presence of TGF-B, SD208 for 48 hours. Data presented as percentage of cells testing positive for α-SMA expression. No significant difference was seen in α-SMA expression in any of the drugged conditions (p = > 0.9)

Data presented as mean ± S.E.M. N = 3 plates, 18 wells, nested one-way ANOVA compared with control.


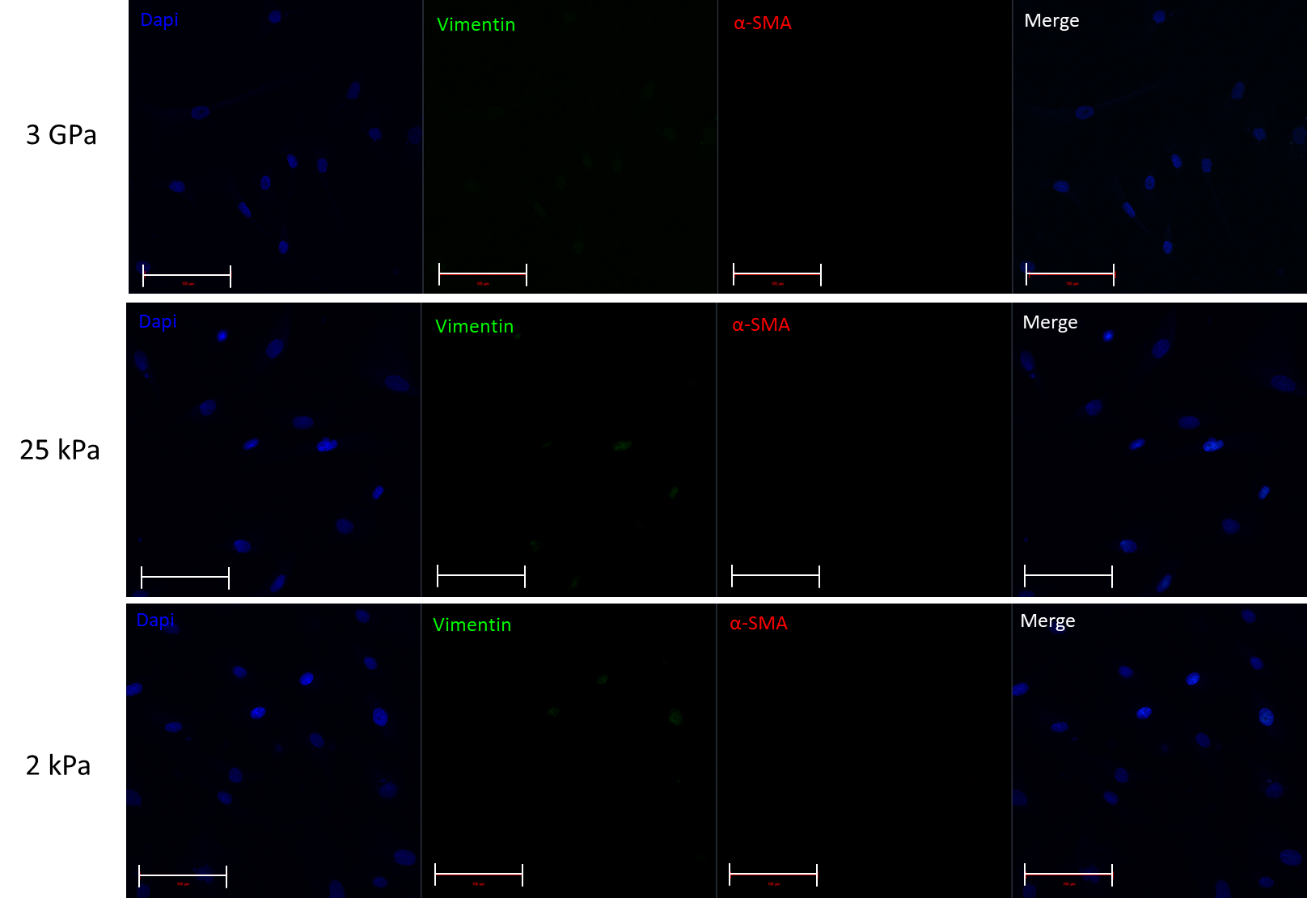


Figure S3. Representative images for secondary antibody only negative controls in HCFs. Cells were grown on 3 GPa plastic or 25 or 2 kPa substrates for 48 hours. No non-specific background staining is observed for Alexa fluor® 488 (vimentin) or Alexa fluor® 647 (α-SMA). Scale bar is 100 µm.


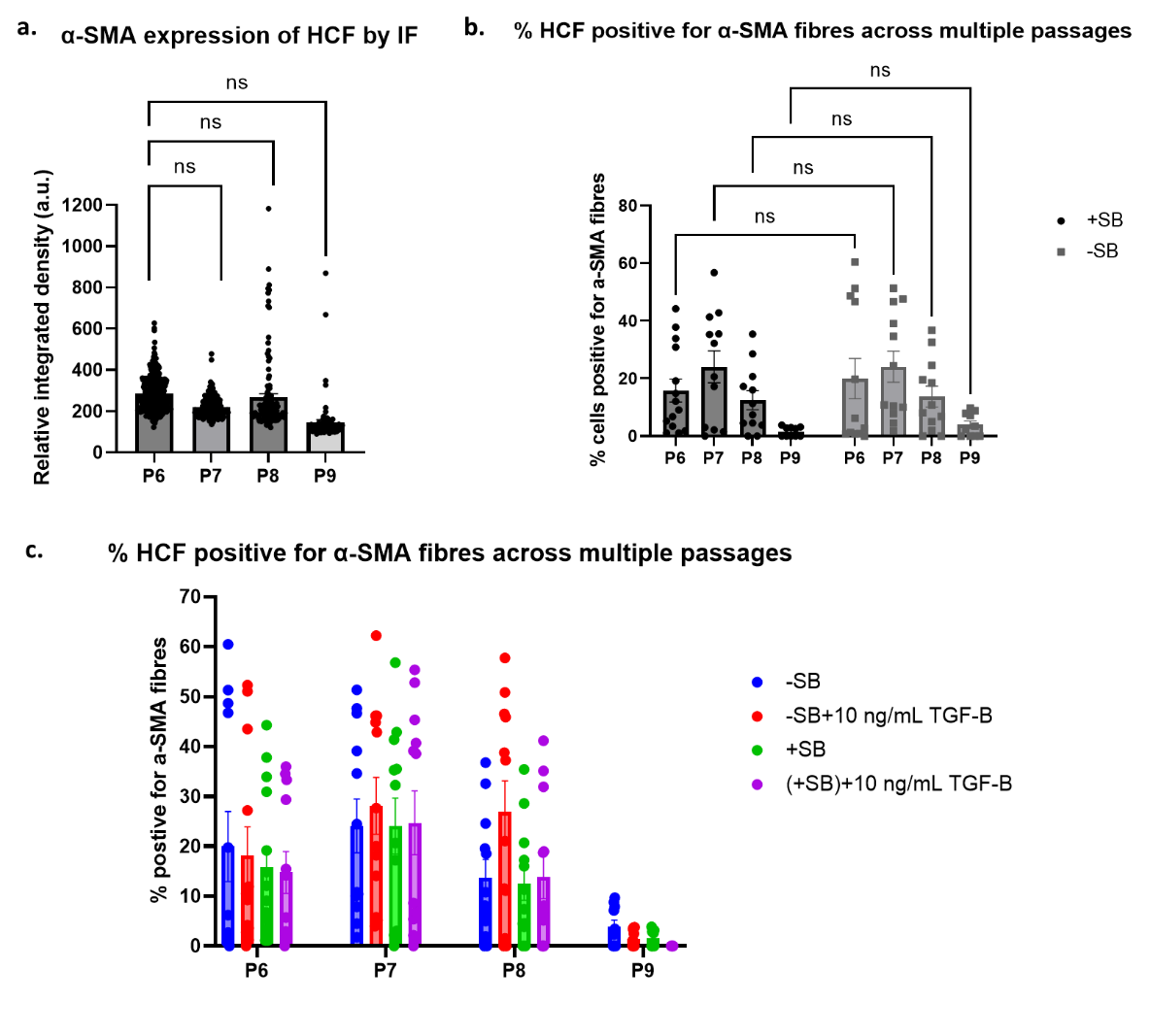


Figure S4. Activation status of human cardiac fibroblasts on stiff plastic across multiple passages. HCF were thawed with and without TGF-β receptor inhibitor SB431542 (SB). At each passage post thaw up to P9 cells were plated and exposed to 10 ng/mL TGF-β for 2 days. a – relative fluorescence staining of α-SMA in individual cells at P6-9 cultured without SB. b – percentage of HCF positive for α-SMA fibrous staining at P6-9 cultured in the presence and absence of SB. c – percentage of HCF positive for α-SMA fibrous staining at P6-9 cultured with and without SB followed by exposure to 10 ng/mL TGF-β for 2 days. Data presented as data ± S.E.M N = 2 cryovials, N = 6 wells, 2 images per well, a = individual cells, b and c = % cells per image


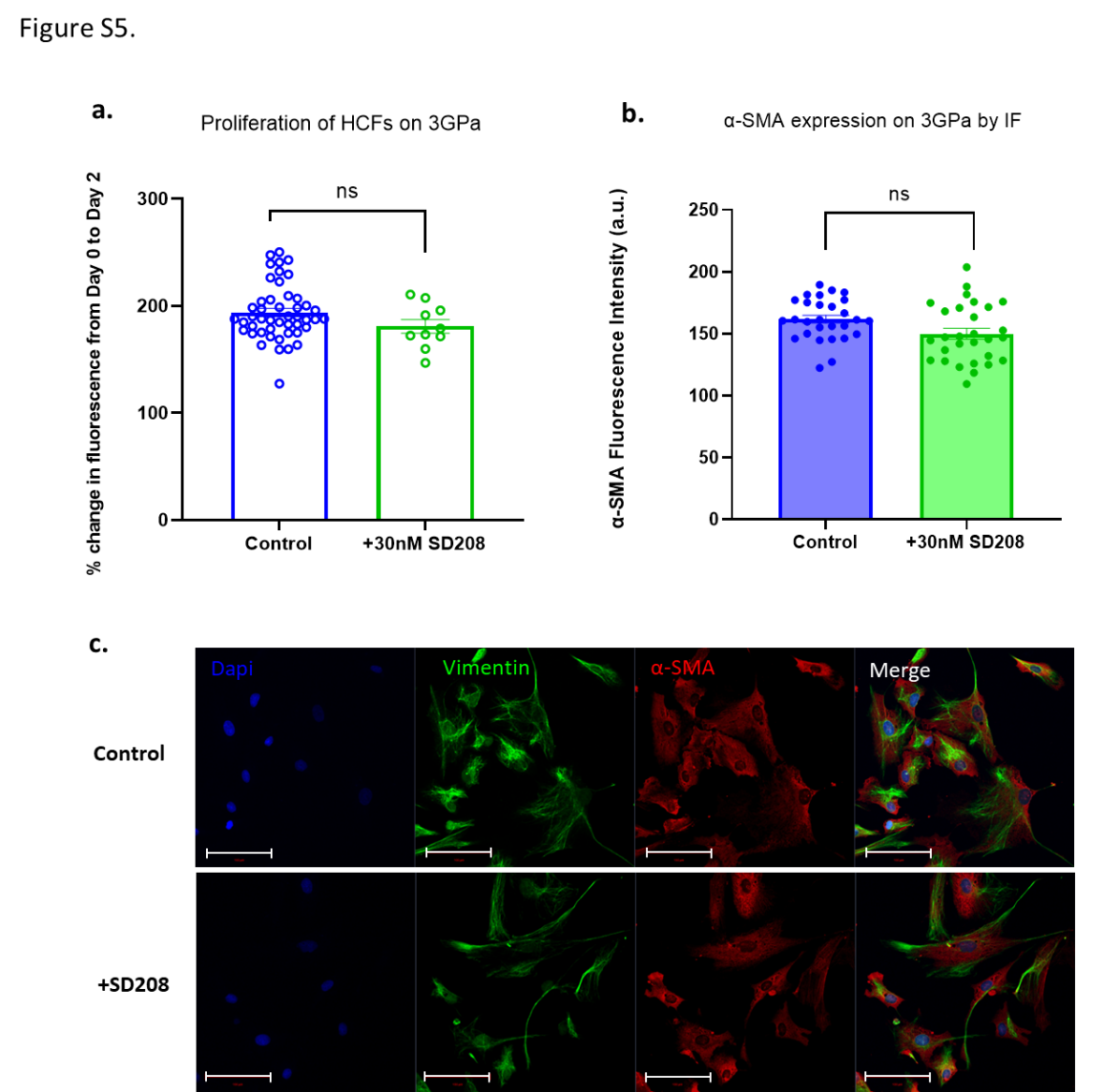


Figure S5. Reversal of myofibroblast activation on stiff plastic using 30 nM SD208. Cells were grown in the presence and absence of 30 nM SD208 for 2 days. a – Proliferation over 48 hours was evaluated as a percentage change from baseline (day 0) in fluorescence by CyQUANT assay. b – Quantification of α-SMA protein expression by immunofluorescence. c – Representative immunofluorescence images for figure b. Scale bar = 100 µm. Data presented as mean ± S.E.M. N = 4 plates, 6+ wells per plate (a), 3 experiments, 3 images per condition (b). nested one-way ANOVA (a, b) compared with undrugged control. Significance was defined by p < 0.05.


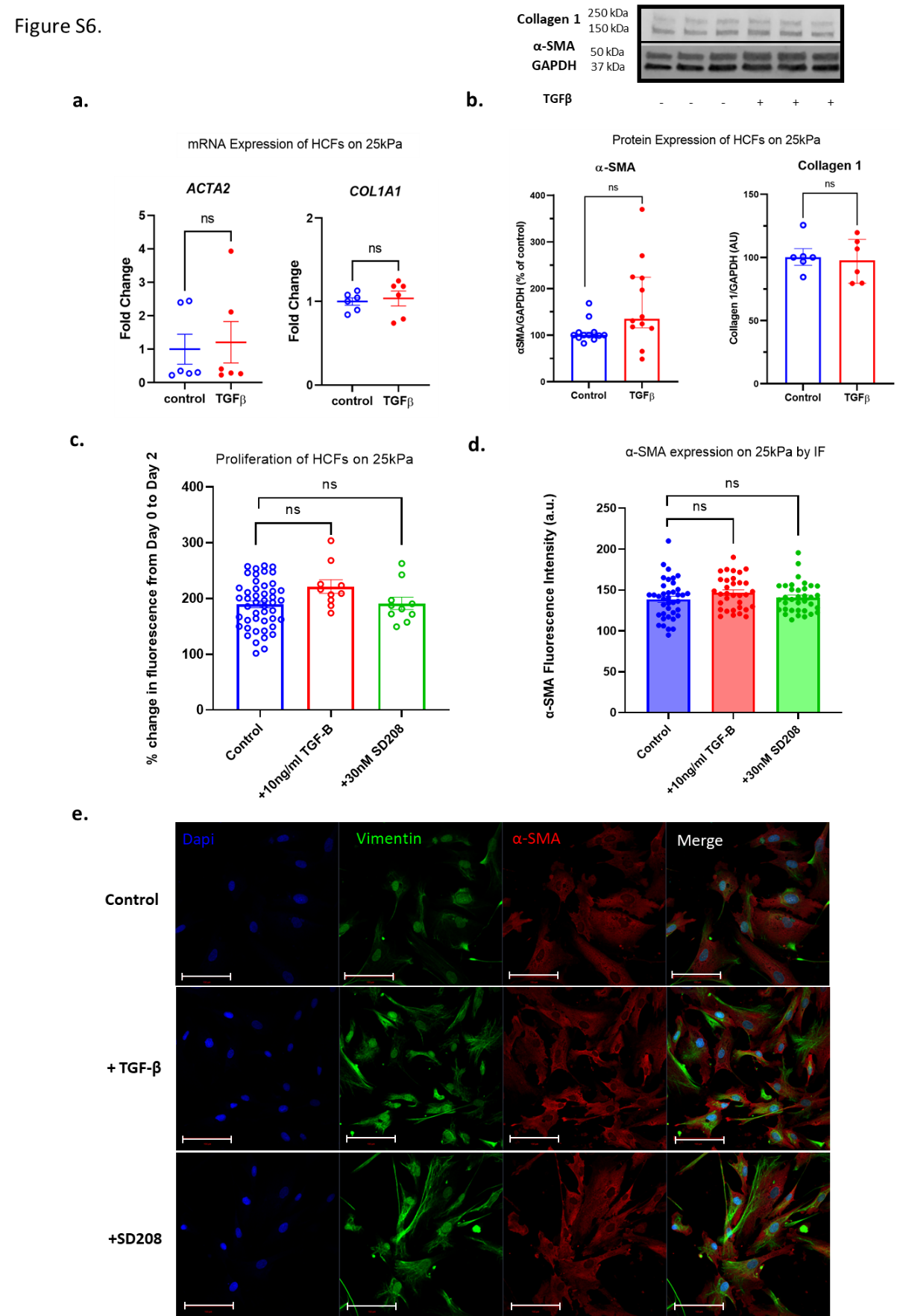


Figure S6. Activation status and proliferation of human cardiac fibroblasts on 25 kPa culture plates. Cells were grown in the presence and absence of TGF-β and/or SD208 for 4 days. a – Activation of HCF as measured by RT-PCR for ACTA2 and COL1A1. b – Activation status of HCF as determined by western blotting of α-SMA and collagen 1 protein. c – Proliferation over 48 hours was evaluated as a percentage change in fluorescence from baseline (day 0) by CyQUANT assay. d – α-SMA expression by immunofluorescence. e – Representative immunofluorescence images for figure d. Scale bar = 100 µm. Data presented as mean ± S.E.M. N = 6 (a), 6-12 (b); 4 plates, 6+ wells per plate (c), 3 experiments, 3 images per condition (d). t-test (a, b) and nested one-way ANOVA (c, d) compared with undrugged control. Significance was defined as p < 0.05.


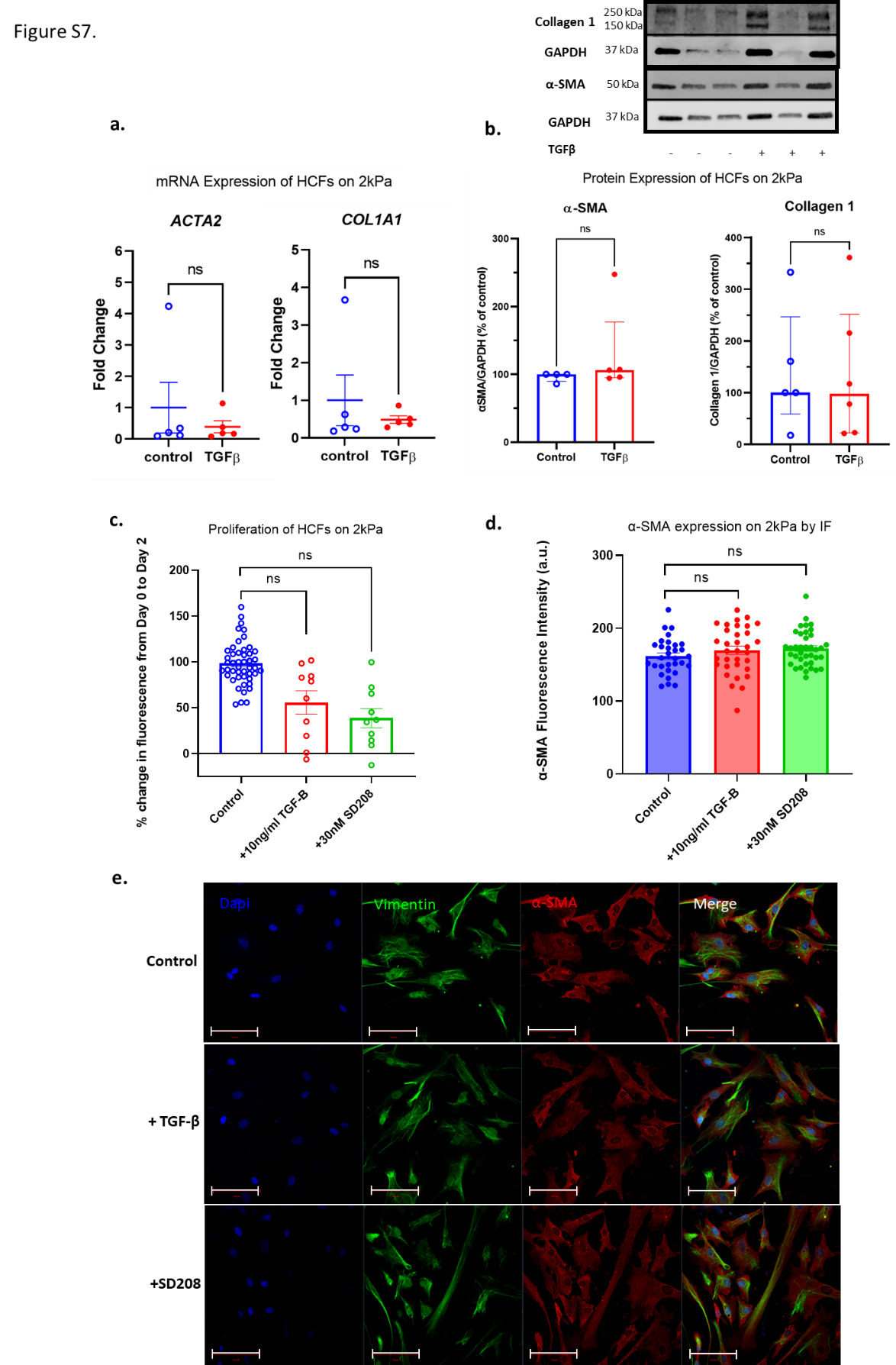


Figure S7. Activation status and proliferation of human cardiac fibroblasts on 2 kPa culture plates. Cells were grown in the presence and absence of TGF-β and/or SD208 for four days. a – Activation of HCF as detected by RT-PCR for ACTA2 and COL1A1. b – Activation status of HCF as determined by western blotting for α-SMA and collagen 1 protein. c – Proliferation over 48 hours was evaluated as a percentage change in fluorescence from baseline (day 0) by CyQUANT assay. d – α-SMA expression by immunofluorescence. e – Representative immunofluorescence images for figure d. Scale bar = 100 µm. Data presented as mean ± S.E.M. N = 5 (a), 4-5 (b); 4 plates, 6+ wells per plate (c), 3 experiments, 3 images per condition (d). t-test (a, b) and nested one-way ANOVA (c, d) compared with undrugged control. Significance was defined as p < 0.05.


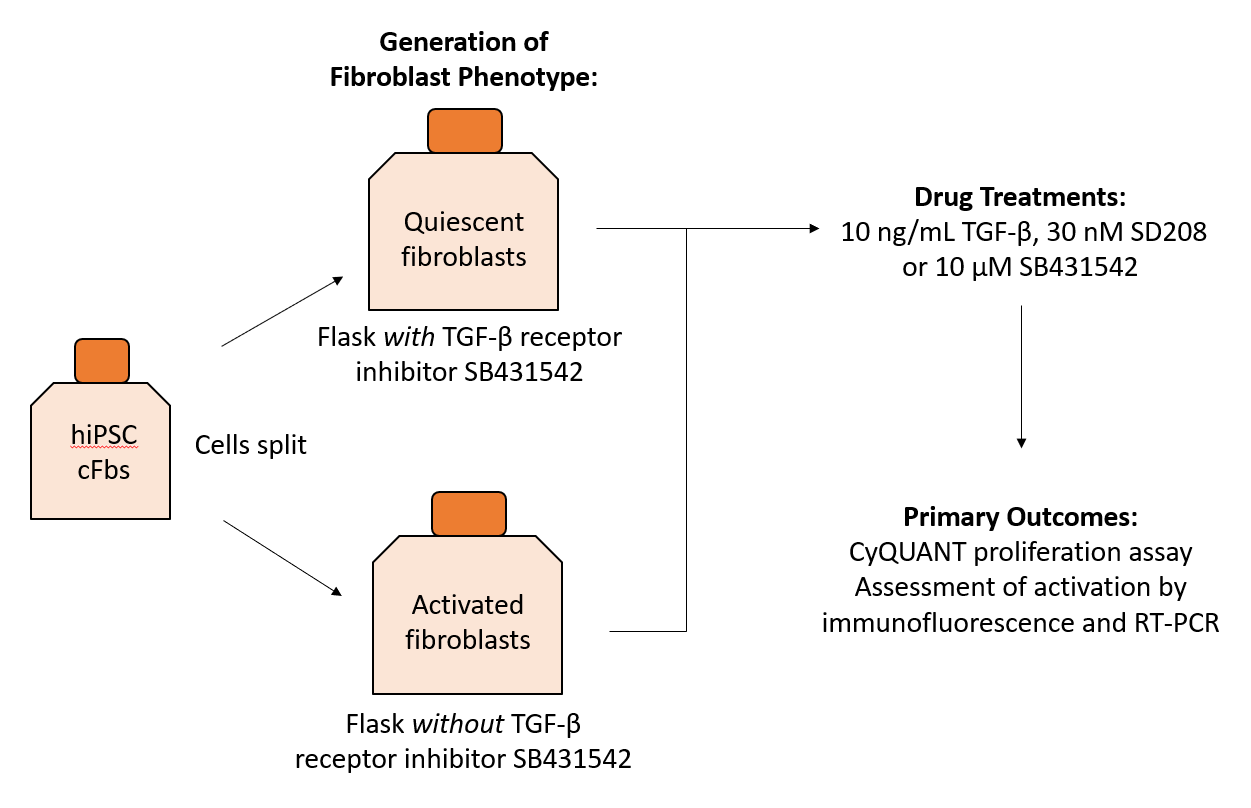


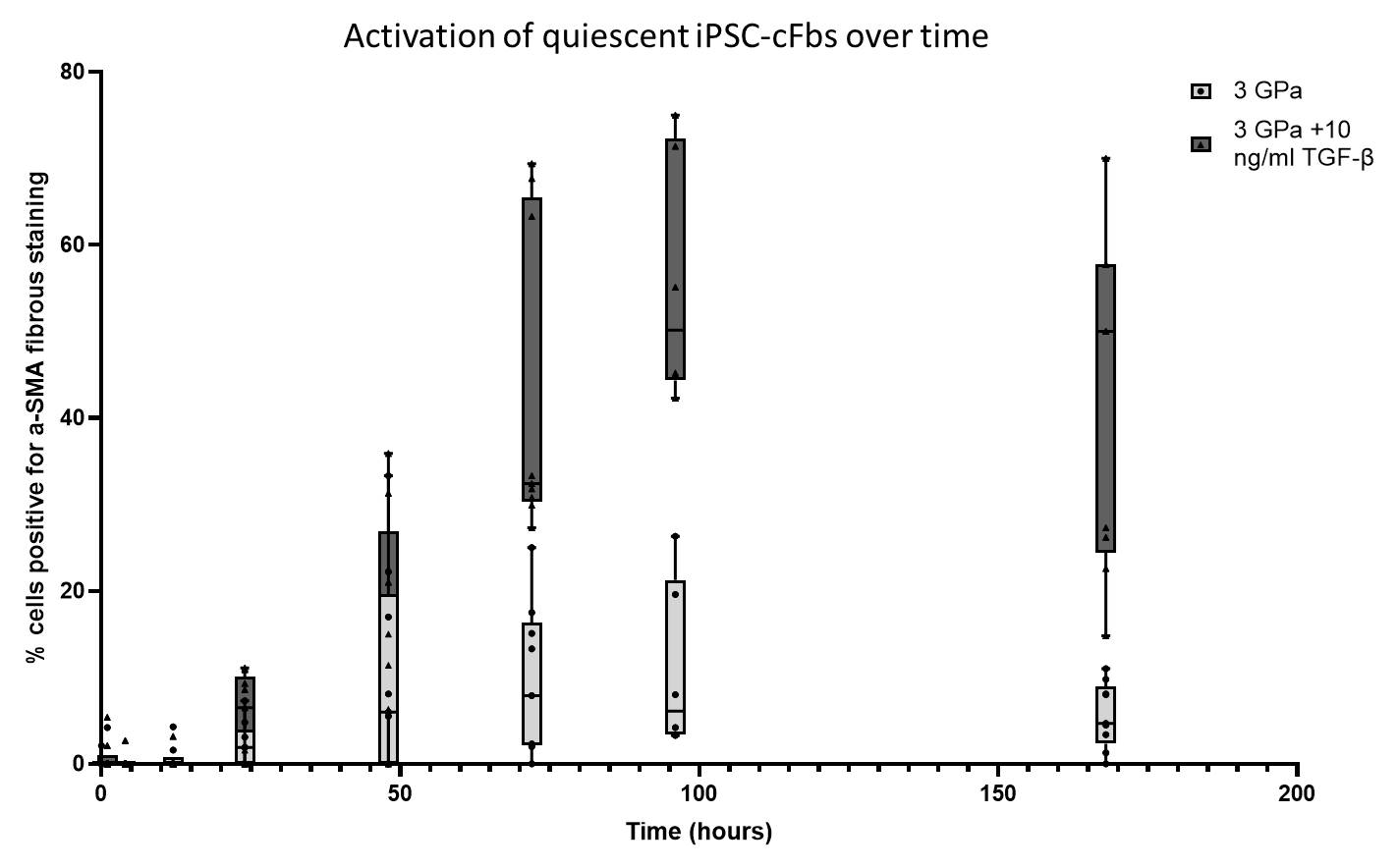


**Figure S9**. **Activation status of quiescent iPSC-cFBs cultured on stiff substrates over 7 days** α-SMA staining in quiescent iPSC-cFbs was assessed over a 7 day time period following culture on 3 GPa substrates, and in the presence of TGF-β. The percentage of cells in a field of view staining positive for α-SMA fibres was recorded. Representative images shown in Figure 9. Data presented as minimum to maximum *N* = 3 wells, 3 images per well.

**Figure S8. Schematic of experimental process for human induced pluripotent stem cell derived cardiac fibroblasts (hiPSC-cFbs).** Cells were grown for 2 weeks in the presence or absence of 10 µM SB431542 to generate quiescent and activated fibroblasts, respectively. hiPSC-cFbs were subjected to further treatments of 10 ng/ml TGF-β, 30 nM SD208 or 10 µM SB431542 for 2-4 days.


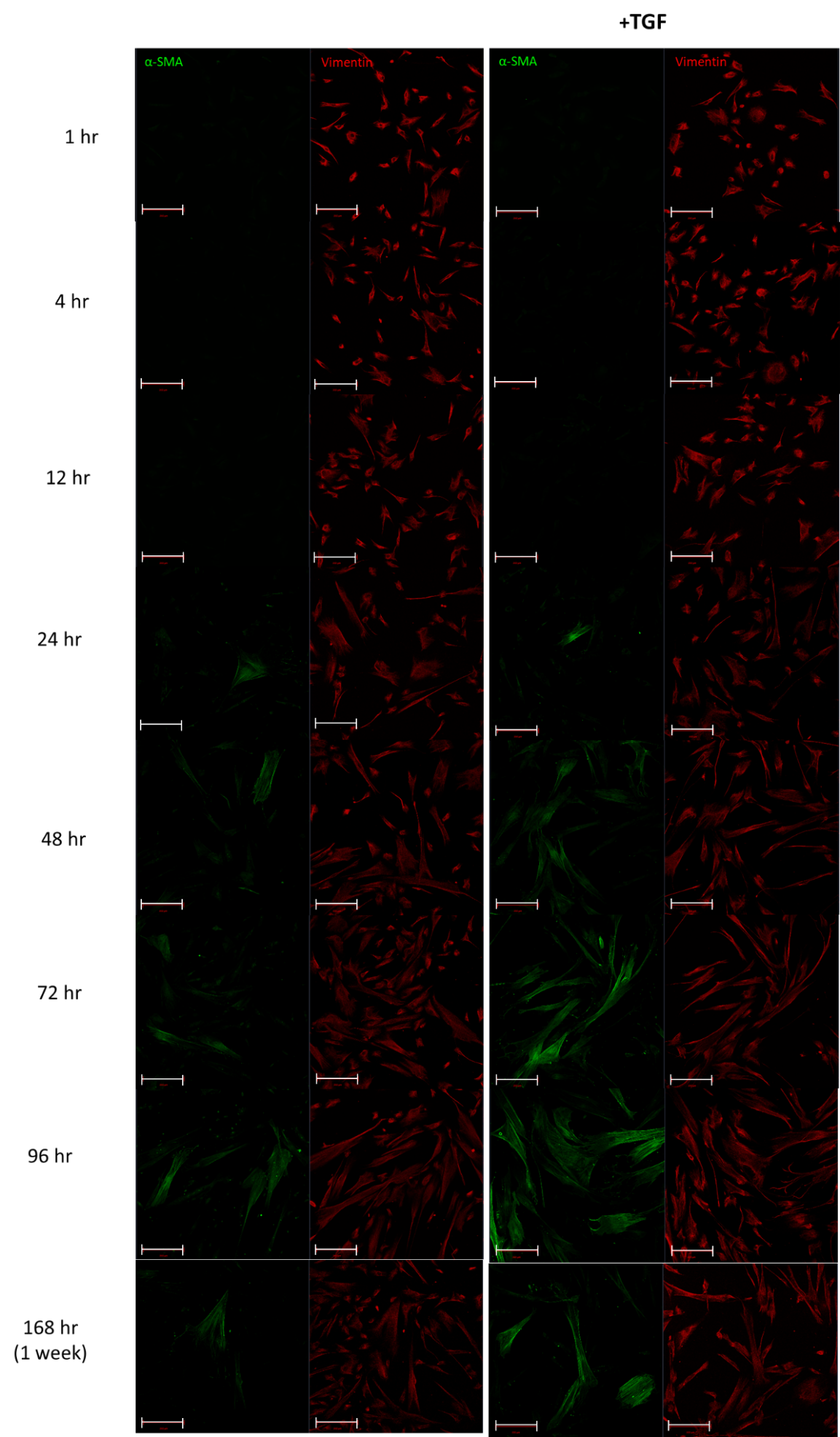


**Figure S10.** **Representative immunofluorescence images for Figure 8**. α-SMA staining in iPSC-cFbs over a 1 week time period in the presence and absence of 10 ng/mL TGF-β. On the left are cells grown without TGF-β and on the right are cells grown with 10 ng/mL TGF-β. α-SMA in green and vimentin, as a marker for cardiac fibroblasts, is in red. Scale bar is 200 µm.


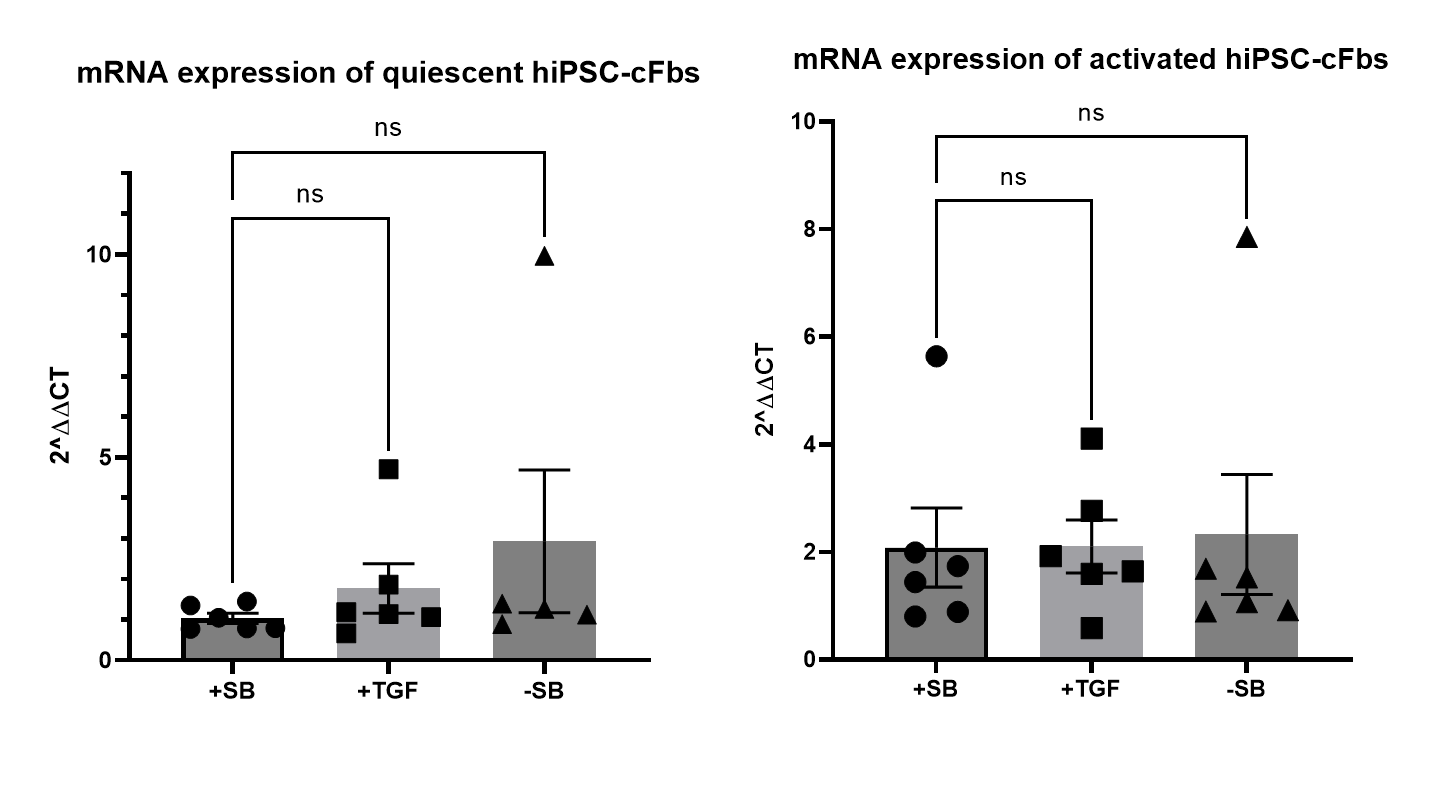


Figure S11. TGF-β receptor 1 expression in activated hiPSC-cFbs in response to culture without SB followed by 4-day exposure to 10 ng/mL TGF-β. Relative mRNA expression of TGF-β receptor 1 as determined by RT-PCR following 4 days in culture with or without SB and TGF-β. Data presented as mean ± S.E.M. N=5-6 wells, p > 0.5

Fold Change
