# Supplementary material for: Chronic activation of human cardiac fibroblasts in vitro attenuates the reversibility of the myofibroblast phenotype
Source: Sci Rep. 2023 Jul 26;13:12137. doi: 10.1038/s41598-023-39369-y (PMC10372150; doi:10.1038/s41598-023-39369-y)
Supplement: Supplementary file 2 — Supplementary Information. [file 41598_2023_39369_MOESM2_ESM.docx]

RAW Western Blots

Images exported from Licor Odyssey Fc were quantified using Licor Image Studio Lite v5.2 analysis software.

**Figure 3B 3GPa**

Membrane A


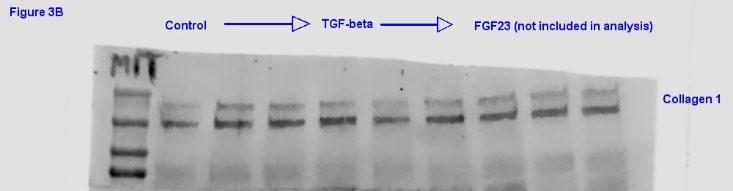


250 kDa

150 kDa

100 kDa

75 kDa


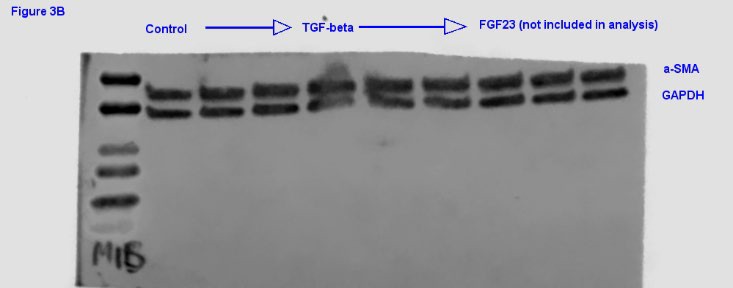


50 kDa

37 kDa

25 kDa

20 kDa

15 kDa

10 kDa

250 kDa

150 kDa

100 kDa

75 kDa

Membrane B


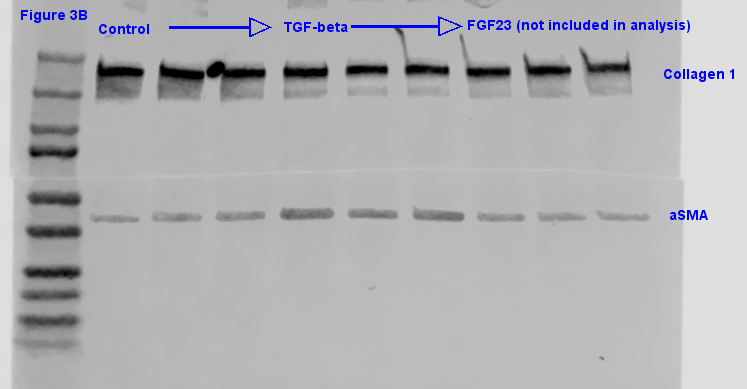


50 kDa

37 kDa

25 kDa

20 kDa

15 kDa

50 kDa

37 kDa

25 kDa

20 kDa

15 kDa

10 kDa


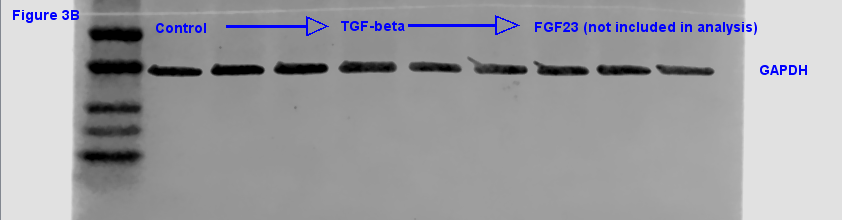


Membrane C

50 kDa

37 kDa

25 kDa

20 kDa

15 kDa

10 kDa


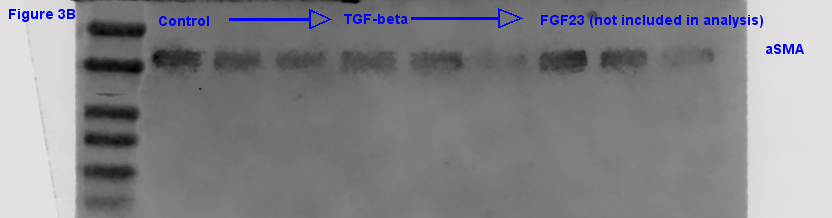


50 kDa

37 kDa

25 kDa

20 kDa

15 kDa

**
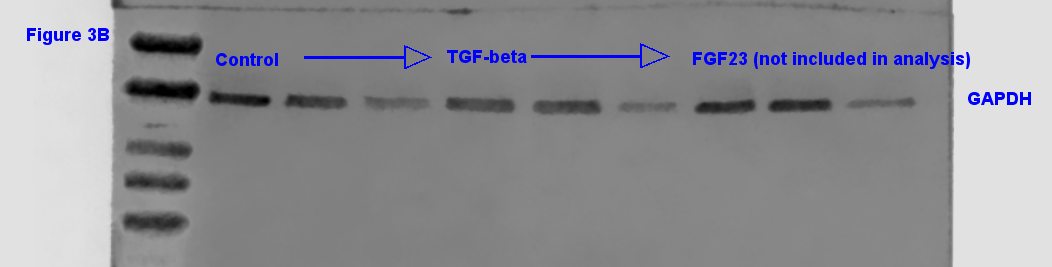
**

**Figure 4B 25kPa**

Membrane A


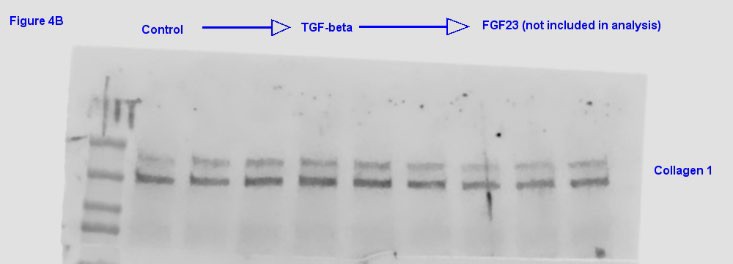


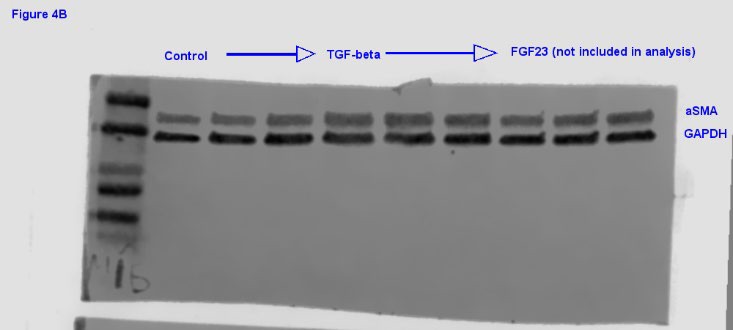


50 kDa

37 kDa

25 kDa

20 kDa

15 kDa

10 kDa

250 kDa

150 kDa

100 kDa

75 kDa

Membrane B


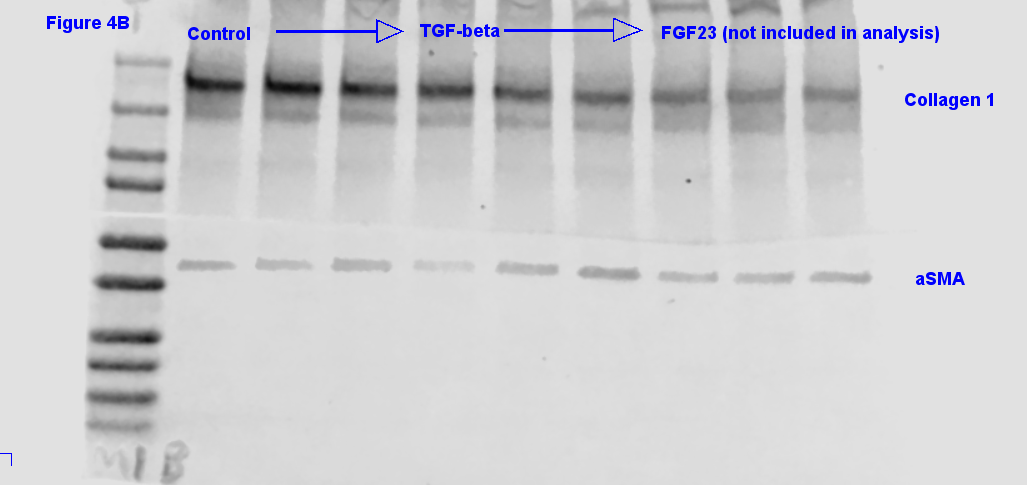


50 kDa

37 kDa

25 kDa

20 kDa

15 kDa

10 kDa

250 kDa

150 kDa

100 kDa

75 kDa

50 kDa

37 kDa

25 kDa

20 kDa

15 kDa

10 kDa


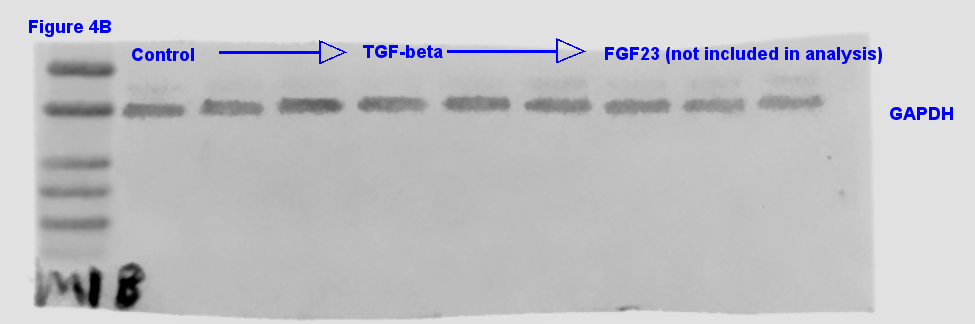


Membrane C


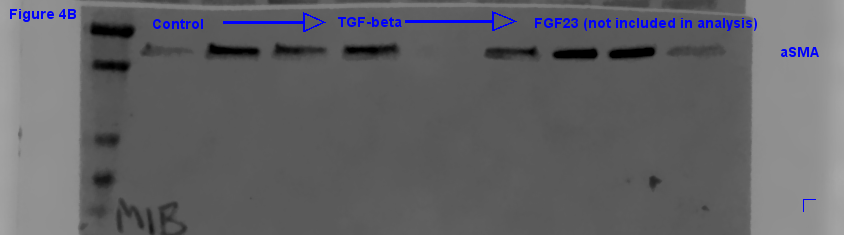


50 kDa

37 kDa

25 kDa

20 kDa

15 kDa


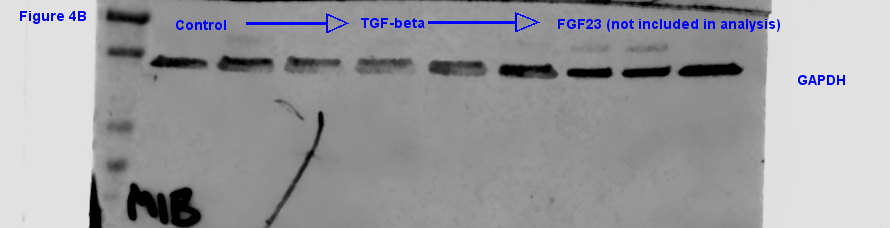


50 kDa

37 kDa

25 kDa

20 kDa

15 kDa

**Figure 4B 25kPa continued**

Membrane D


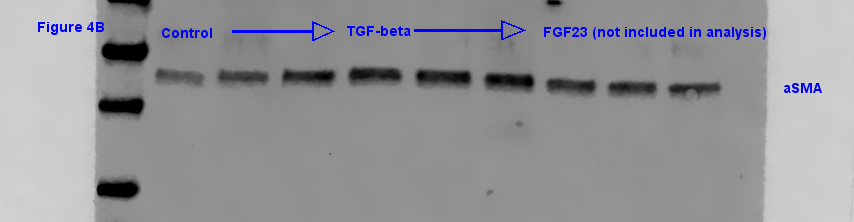


50 kDa

37 kDa

20 kDa


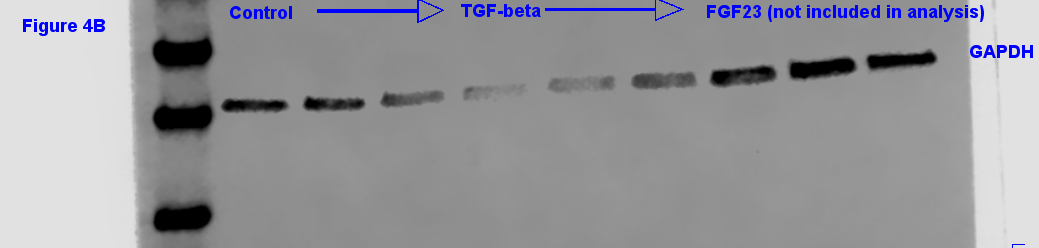


50 kDa

37 kDa

20 kDa

**Figure 5B 2kPa**

Membrane A


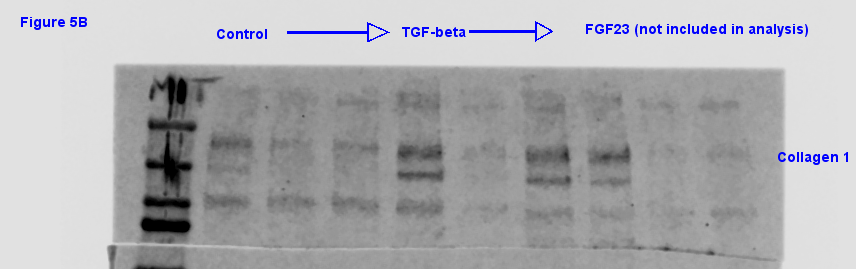


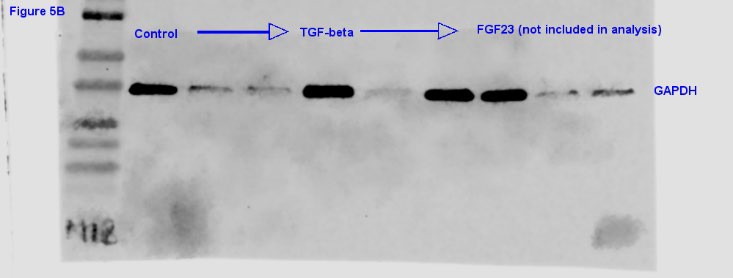


50 kDa

37 kDa

25 kDa

20 kDa

15 kDa

10 kDa

250 kDa

150 kDa

100 kDa

75 kDa

Membrane B


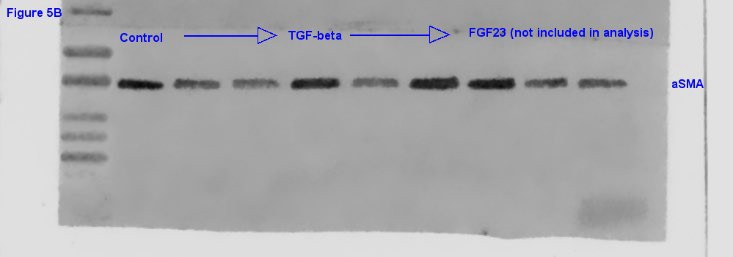


50 kDa

37 kDa

25 kDa

20 kDa

15 kDa

10 kDa


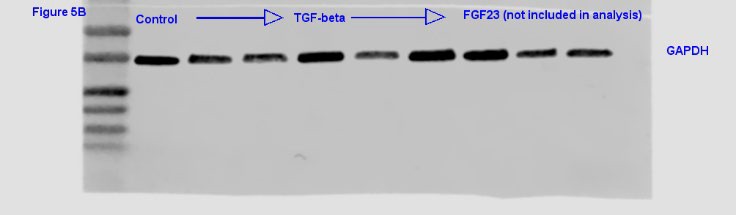


50 kDa

37 kDa

25 kDa

20 kDa

15 kDa

10 kDa

Membrane C


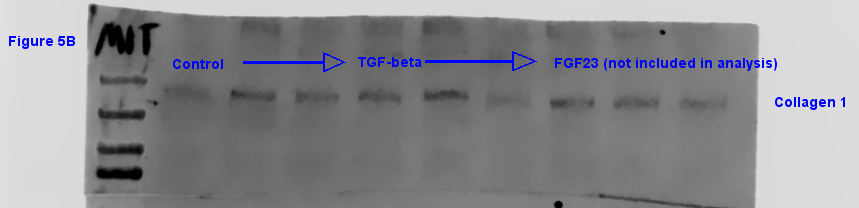


250 kDa

150 kDa

100 kDa

75 kDa


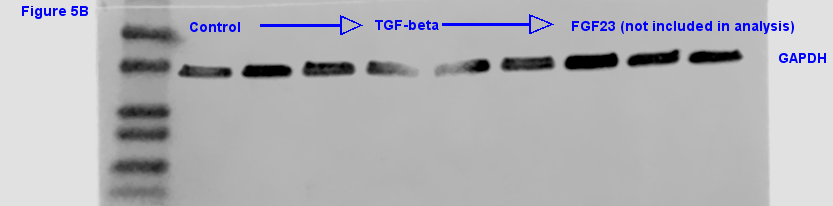


50 kDa

37 kDa

25 kDa

20 kDa

15 kDa

10 kDa

Membrane D


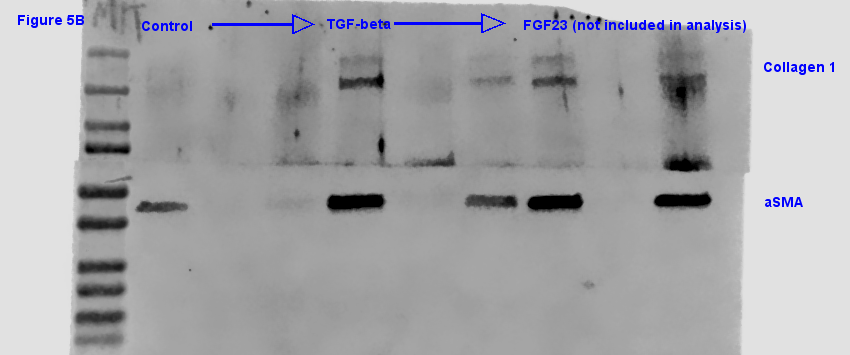


50 kDa

37 kDa

25 kDa

20 kDa

15 kDa

10 kDa

250 kDa

150 kDa

100 kDa

75 kDa


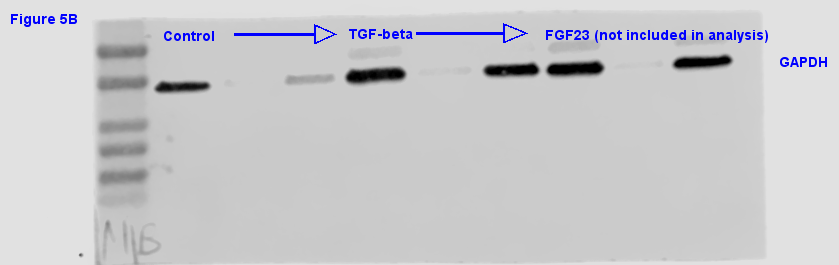


50 kDa

37 kDa

25 kDa

20 kDa

15 kDa

10 kDa
